# Microglial-mediated PDGF-CC activation increases cerebrovascular permeability during ischemic stroke

**DOI:** 10.1007/s00401-017-1749-z

**Published:** 2017-07-19

**Authors:** Enming Joseph Su, Chunzhang Cao, Linda Fredriksson, Ingrid Nilsson, Christina Stefanitsch, Tamara K. Stevenson, Juanjuan Zhao, Margret Ragsdale, Yu-Yo Sun, Manuel Yepes, Chia-Yi Kuan, Ulf Eriksson, Dudley K. Strickland, Daniel A. Lawrence, Li Zhang

**Affiliations:** 1 Division of Cardiovascular Medicine, Department of Internal Medicine, University of Michigan Medical School, 7301 MSRB III, 1150 W. Medical Center Dr., Ann Arbor, MI 48109-0644 USA; 2 Department of Physiology, Center for Vascular and Inflammatory Diseases, University of Maryland School of Medicine, BioPark-1, Room 211, 800 W. Baltimore Street, Baltimore, MD 21201 USA; 3 Division of Vascular Biology, Department of Medical Biochemistry and Biophysics, Karolinska Institutet, Scheeles v. 2, 17177 Stockholm, Sweden; 4 Department of Molecular and Integrative Physiology, University of Michigan Medical School, Ann Arbor, MI USA; 5 Department of Pediatrics, The Center for Neurodegenerative Disease, Emory University, Atlanta, GA USA; 6 Department of Neurology, Emory University, Atlanta, GA USA

**Keywords:** Stroke, Blood–brain barrier, Platelet-derived growth factor-CC, Tissue plasminogen activator, Mac-1, α_M_β_2_, CD11b/CD18, LRP1

## Abstract

Treatment of acute ischemic stroke with the thrombolytic tissue plasminogen activator (tPA) can significantly improve neurological outcomes; however, thrombolytic therapy is associated with an increased risk of intra-cerebral hemorrhage (ICH). Previously, we demonstrated that during stroke tPA acting on the parenchymal side of the neurovascular unit (NVU) can increase blood–brain barrier (BBB) permeability and ICH through activation of latent platelet-derived growth factor-CC (PDGF-CC) and signaling by the PDGF receptor-α (PDGFRα). However, in vitro, activation of PDGF-CC by tPA is very inefficient and the mechanism of PDGF-CC activation in the NVU is not known. Here, we show that the integrin Mac-1, expressed on brain microglia/macrophages (denoted microglia throughout), acts together with the endocytic receptor LRP1 in the NVU to promote tPA-mediated activation of PDGF-CC. Mac-1-deficient mice (Mac-1^−/−^) are protected from tPA-induced BBB permeability but not from permeability induced by intracerebroventricular injection of active PDGF-CC. Immunofluorescence analysis demonstrates that Mac-1, LRP1, and the PDGFRα all localize to the NVU of arterioles, and following middle cerebral artery occlusion (MCAO) Mac-1^−/−^ mice show significantly less PDGFRα phosphorylation, BBB permeability, and infarct volume compared to wild-type mice. Bone-marrow transplantation studies indicate that resident CD11b^+^ cells, but not bone-marrow-derived leukocytes, mediate the early activation of PDGF-CC by tPA after MCAO. Finally, using a model of thrombotic stroke with late thrombolysis, we show that wild-type mice have an increased incidence of spontaneous ICH following thrombolysis with tPA 5 h after MCAO, whereas Mac-1^−/−^ mice are resistant to the development of ICH even with late tPA treatment. Together, these results indicate that Mac-1 and LRP1 act as co-factors for the activation of PDGF-CC by tPA in the NVU, and suggest a novel mechanism for tightly regulating PDGFRα signaling in the NVU and controlling BBB permeability.

**Electronic supplementary material:**

The online version of this article (doi:10.1007/s00401-017-1749-z) contains supplementary material, which is available to authorized users.

## Introduction

Stroke is a leading cause of morbidity and the fifth leading cause of mortality in the United States [1]. There are two types of stroke, ischemic and hemorrhagic. The majority of strokes are ischemic, with hemorrhagic stroke accounting for approximately 10–13% of strokes [47]. Hemorrhagic strokes generally have a worse prognosis [32] than ischemic strokes, and hemorrhagic conversion of an ischemic stroke markedly increases stroke severity [24, 29, 39, 64, 65]. The current standard of care for patients with ischemic stroke is thrombolytic therapy with tPA [52]. However, thrombolysis carries a significant risk of intra-cerebral hemorrhage [24, 29, 39, 64, 65], and due in part to this increased risk of hemorrhagic conversion, it is estimated that only 5–7% of ischemic stroke patients receive intravenous tPA, with another 1–2% receiving intra-arterial therapy [34, 52]. The limited use of thrombolytic therapy demands further study of the mechanisms by which tPA leads to an increased risk of hemorrhagic conversion in stroke patients.

tPA is a highly specific serine protease of the fibrinolytic cascade, and we and others have demonstrated that tPA increases BBB permeability after cerebral ischemia, via an LRP1-dependent mechanism [61, 67–69]. tPA is both necessary and sufficient to induce early opening of the BBB after experimental MCAO [61, 69]. This effect requires proteolytically active tPA, but is independent of plasminogen, implying another substrate for tPA [69]. PDGF-CC is a homodimeric member of the PDGF family with a two-domain structure, and tPA cleavage of the amino-terminal CUB domains from latent PDGF-CC generates active PDGF-CC capable of triggering PDGFRα signaling [17, 19]. In the NVU, activation of PDGF-CC leads to loss of BBB integrity and increases the extent of thrombolytic tPA-induced ICH [61]. Blocking this pathway has been shown to significantly improve outcome and reduce ICH in a murine model of ischemic stroke [61]. Importantly, a very recent phase II clinical trial testing the safety of blocking this pathway in patients treated with intravenous thrombolysis after ischemic stroke has demonstrated that the PDGFRα antagonist imatinib is both safe and tolerable, and improves neurological outcomes in these patients [66]. Thus, understanding the mechanism of tPA-mediated PDGF-CC activation within the NVU has important clinical implications and may accelerate the development of adjuvant treatments that could reduce the risk of hemorrhagic complications associated with thrombolytic therapy for ischemic stroke.

An unusual feature of tPA is that unlike most serine proteases, it is produced as an active enzyme rather than a zymogen that requires proteolytic activation [44]. However, the catalytic efficiency of tPA is relatively poor compared to typical serine proteases such as trypsin, which is approximately 1500-fold more efficient than tPA [12, 14]. Thus, the activity of tPA is largely controlled by co-factors, such as fibrin, which can increase tPA’s catalytic efficiency by more than two orders of magnitude [43, 58]. Similarly, the activation of PDGF-CC by purified tPA in vitro is inefficient [19, 61]. In contrast, in vivo tPA is able to rapidly increase BBB permeability in a PDGF-CC/PDGFRα-dependent process, suggesting that there are co-factors within the NVU that facilitate the efficient activation of PDGF-CC by tPA [61, 69]. Two potential candidate co-factors present in the brain are: LRP1, which has been shown to accelerate tPA-mediated cleavage of latent PDGF-CC in cell culture studies [61], and the integrin Mac-1 (also called α_M_β_2_ and CD11b/CD18). Mac-1 is expressed on microglia [5, 13] and has been shown to associate with both tPA and LRP1 on leukocytes [9], and like tPA-deficient mice [68], Mac-1^−/−^ mice are protected from ischemic stroke [60] (Online Resource 1). Together, these studies suggest a possible link between Mac-1, LRP1, and tPA-mediated PDGF-CC activation in the NVU.

In the present study, we test the hypothesis that Mac-1 and LRP1 coordinate to enhance tPA-mediated PDGF-CC activation by acting as co-factors for tPA in the NVU. Using both a cell-based PDGFRα activation assay and a photothrombotic model of ischemic stroke, we show that both Mac-1 and LRP1 are required for efficient activation of PDGF-CC by tPA and the subsequent phosphorylation of the PDGFRα. Identification of these key interactions is essential for understanding the regulation of BBB integrity by tPA during cerebral ischemia and may provide novel opportunities to improve the safety of thrombolytic treatment for ischemic stroke.

## Methods

### Mice

Wild-type (WT), Mac-1^−/−^ [60], CX3CR1^GFP^/CCR2^RFP^ (R/G) [31, 57], *Pdgfra H2B*-*eGFP* (*Pdgfra* ^*GFP/*+^) [25], and the B6.129S7-Gt(ROSA)26Sor/J (ROSA) [22] mice were all in the C57BL/6J background and used at ~8–13 weeks (20–22 g). WT, R/G, and ROSA mice were purchased from the Jackson Laboratory. Mac-1^−*/*−^ mice were kindly provided by Dr. Christie M. Ballantyne, Baylor College of Medicine (Houston, TX, USA), and the *Pdgfra* ^*GFP/*+^ mice by Dr. Philippe M. Soriano, Icahn School of Medicine at Mount Sinai (New York, NY, USA). Mac-1^−/−^ ROSA mice were generated by crossing ROSA with Mac-1^−/−^ mice. The bitransgenic R/G mice are heterozygous for CX3CR1^GFP^/CCR2^RFP^ expressing GFP in microglia and macrophages under the control of the CX3CR1 promoter and RFP in monocytes and macrophages under the control of the CCR2 promoter. These bitransgenic knock-in mice are used as heterozygous mice to avoid deficiency of CCR2 or CX3CR1 gene expression. The *Pdgfra* ^*GFP/*+^ express a nuclear GFP signal in cells, where the *Pdgfra* promoter is/has been active. All mice were housed in a pathogen-free facility and all procedures were performed in accordance with the local welfare legislation and approved by the Institutional Animal Care and Use Committees at the University of Michigan, University of Maryland, Emory University and Karolinska Institutet.

### Preparation of primary microglia

Cortices from 1- to 3-day-old WT and Mac-1^−/−^ male and female mice were dissected and then treated with 0.4% trypsin for 20 min at 37 °C. Cells were plated onto poly-d-lysine-coated 75-cm^2^ tissue culture flasks and cultured in 10% FBS in DMEM. After 2–3 weeks, microglia were removed by orbital shaking (200 rpm) with the addition of 12 mM lidocaine (Sigma) for 20 min at 37 °C. Microglia were collected by centrifugation and maintained in poly-d-lysine-coated tissue culture dishes. The purity of the microglial preparation was verified by flow cytometry.

### Cell-based receptor activation system to monitor PDGF-CC activation

Recombinant full-length latent PDGF-CC was expressed in Sf9 cells. The latent PDGF-CC was then incubated for 90 min with or without 100 nM tPA in a 6-well tissue culture plate, with or without 5 × 10^6^ primary microglia or BV2 cells (an immortalized murine microglial cell line expressing Mac-1 and LRP1, Online Resource 2). In some experiments, specific antagonists of Mac-1 (NIF; 100 nM), tPA (anti-murine-tPA mAb H27B6; 20 µg/ml or PAI-1; 70 nM), plasmin (aprotinin; 3kIU/ml), LRP1 (RAP; 200 nM or rabbit anti-LRP1 antibody 2629; 100 µg/ml), or their corresponding control IgGs were added. The cell media were then collected and evaluated for the appearance of active PDGF-CC by SDS-PAGE followed by immunoblotting with goat anti-PDGF-CC antibody C-17 (Santa Cruz), or for activation of PDGF-CC downstream signaling activity using porcine aortic endothelial cells stably expressing recombinant PDGFRα but not the PDGFRβ (PAE-α) [16]. PAE-α cells were cultivated in 100 mm tissue culture Petri dishes and were incubated with 1 ml of the cell media containing the above PDGF-CC activation mixture or recombinant active PDGF-CC core protein (as a control) in 5 ml DMEM on ice for 40 min. The cells were lysed in 500 μl of RIPA lysis buffer (EMD Millipore, Billerica, MA, USA) containing a cocktail of protease inhibitors and phosphatase inhibitors (Cell Signaling Technology, Danvers, MA, USA). The cell lysates were then subjected to SDS-PAGE. Total and phospho-PDGFRα were determined by immunoblotting using a rabbit anti-PDGFRα (Cell Signaling) for total PDGFRα and a rabbit anti-phospho-PDGFRα (Tyr 720) (Santa Cruz Biotechnology, Dallas, Texas, USA) for phospho-PDGFRα. The degree of PDGF-CC activation was determined based on the ratio between phosphorylated and total PDGFRα.

### PDGF-CC activation assay using purified proteins

Preparations of the soluble form of LRP1 (sLRP1) and full-length Mac-1 were based on our published methods [10, 53]. The latent PDGF-CC is incubated with 100 nM tPA in 100 µl of DMEM in a 96-well plate, with the addition of 65 nM purified Mac-1, 10 nM sLRP1 or both Mac-1 and sLRP1 at 37 °C for 90 min. Generation of active PDGF-CC was quantified as described above except that 60 mm tissue culture dishes were used for the PDGFRα phosphorylation assays.

### Knock-down of Mac-1 or LRP1 expression in BV2 cells

BV2 cells were transfected with Mission lentiviral particles (Sigma-Aldrich) according to production instructions. For each gene, five different shRNAs (see Online Resource 3) were tested in transient experiments. Their abilities to silence gene expression were determined by qRT-PCR and immunoblotting. The two most potent shRNAs, including 5′CCGGGCCTTGTGTCATGGCTTCAATCTCGAGATTGAAGCCATGACACA-AGGCTTTTTG3′ and 5′CGGGCAGCCAGATTGGCTCTTATTCTCGAGAATAAGA-GCCAATCTGGCTGCTTTTTG3′ (for Mac-1) and 5′CCGGGCTGAACACATTCTTTGGTA-ACTCGAGTTACCAAAGAATGTGTTCAGCTTTTTG3′ and 5′CCGGCCTACCTACA-AGATGTATGAACTCGAGTTCATACATCTTGTAGGTAGGTTTTTG3′ (for LRP1), were used subsequently to knock-down expression of Mac-1 and LRP1 in BV2 cells, respectively. Stable cell lines were established by the addition of 5 µg/ml puromycin. After 20 days, stable cells were pooled and their expression of Mac-1 and LRP1 was analyzed by flow cytometry and immunoblot. These stable BV2 cells were used in PDGF-CC activation assays.

### Immunostaining and confocal microscopy

Tissue preparation for sectioning and immunostaining was conducted using standard protocols. In short, mice were anesthetized with isoflurane and then transcardially perfused with phosphate-buffered saline (PBS) followed by perfusion with 4% paraformaldehyde (PFA) in PBS. The brains were removed, post-fixed in 4% PFA 1 h at room temperature (RT), and submerged in 30% sucrose. The brains were thereafter cryopreserved in OCT and sectioned in a cryostat. Alternatively, the brains were sectioned using a vibratome. Following immunofluorescent staining, the sections were mounted using ProLong Gold Antifade reagent (P36930, Life Technologies, Molecular Probes, Grand Island, NY, USA). If not stated otherwise, images were acquired at RT with a Zeiss LSM700 confocal microscope and the ZEN 2009 software (Carl Zeiss Microimaging GmbH, Jena, Germany). The images shown are representative of the respective staining and were processed and analyzed using the Volocity 3D image analysis software (PerkinElmer, Waltham, MA, USA), Photoshop CS5 (Adobe, San Jose, CA, USA), or ImageJ64 (National Institutes of Health, Bethesda, MD, USA).

Brains from adult naïve WT mice were used for co-localization of CD11b, LRP1, NG2, GFAP, and PDGFRα in the neurovascular unit. Vibratome sections (50 μm sections) were stained free-floating in 24-well plates. The sections were permeabilized with 1% BSA in 0.5% TritonX-100/PBS over night at 4 °C followed by incubation with primary antibodies (1:200) in blocking solution 0.5% BSA in 0.25% TritonX-100/PBS over night at 4 °C. The specific primary antibodies used were: rat anti-CD11b (550282, BD Biosciences, Franklin Lakes, NJ, USA), rabbit anti-LRP1 (#Rab2629, made In House Prof. DK Strickland), rabbit anti-NG2 (AB5320, Merck/Millipore), rabbit anti-GFAP (Z0334, Agilent Technologies/Dako), and goat anti-PDGFRα (AF1062; R&D Systems, Minneapolis, MN, USA). Goat or rat anti-CD31 antibodies were used to visualize endothelial cells (AF3628, R&D Systems, UK; and 553370, BD Biosciences, respectively). After thorough wash, the sections were incubated with appropriate Alexa Flour^®^-conjugated secondary antibodies and Alexa Flour^®^-conjugated streptavidin (Life Technologies, Molecular Probes) in blocking solution 0.5% BSA in 0.25% TritonX-100/PBS over night at 4 °C, then washed and mounted.

For detection of reactive microglia and in vivo phosphorylation of PDGFRα following ischemic stroke, MCAO was induced in WT, Mac-1^−*/*−^, and *Pdgfra* ^*GFP/*+^ mice. At 6 and 24 h after MCAO, the brains were harvested, cryopreserved in OCT, and sectioned. The sections were blocked in TNB buffer (TSA biotin system, NEL700A001KT, Perkin Elmer), followed by incubation over night at 4 °C with primary antibodies diluted in TNB buffer. The specific primary antibodies used were: rabbit anti-Iba1 (1:250, 019-19741, Wako Pure Chemical Industries Ltd., Osaka, Japan), rabbit anti-phospho-tyrosine (pY) 754 or -1018 specific PDGFRα (1:200, pY754 #2992 and pY1018 #4547; Cell Signaling, Danvers, MA, USA). Goat anti-CD31 or goat anti-podocalyxin antibodies were used to visualize endothelial cells (1:250; R&D Systems, UK). Appropriate Alexa Flour^®^-conjugated secondary antibodies (Life Technologies, Molecular Probes) were used to detect the bound primary antibodies.

Microglia activation was quantified using PFA perfusion-fixed brains. Iba1-stained brain sections (12 μm) from Mac-1^−*/*−^ and WT mice (*n* = 3 per genotype and timepoint) were analyzed by two independent investigators blinded to the study group. The individual observations are based on analysis of five fields of view taken in the penumbra and contralateral cortex of each animal with the 20× objective using the Zeiss LSM700 confocal microscope. Cell body area was quantified using Volocity 3D and vessel association using the NIH Image J software. For quantification of PDGFRα activation in situ, unfixed brains from Mac-1^−*/*−^ (*n* = 8) and WT (*n* = 6) mice subjected to 6 h of MCAO were used. Images were captured with a Nikon Eclipse TE800 upright microscope (Nikon, Tokyo, Japan). Quantification was performed using the NIH Image J software and the area of antibody immunoreactivity was determined from four to seven images per animal and normalized to WT control. *n* indicates the number of individual mice used in the study. The result from all the fields of view in a given animal was averaged to obtain the value for that individual. Individual values and group mean ± SEM are shown.

The specificity of the phospho-tyrosine specific PDGFRα antibodies was confirmed by Western blot analysis (Online Resource 4). Briefly, C6 glioma cells (CCL-107, ATCC) expressing PDGFRα, and PAE cells expressing PDGFRβ but not PDGFRα (PAE-β) [16], were utilized. Cells were starved for 6 h in F12 medium containing 0.5% serum and thereafter acutely stimulated with 20 ng/ml active PDGF-CC (produced in house) or PDGF-DD (1159-SB; R&D Systems, Minneapolis, MN, USA) for 10 min at 37 °C to selectively induce PDGFRα and PDGFRβ receptor phosphorylation, respectively. Non-stimulated cells served as controls. Cells were lysed directly in SDS sample buffer and the proteins separated by SDS-PAGE and transferred to a nitrocellulose membrane. After blocking, membranes were incubated with the pY754 or pY1018 specific PDGFRα antibodies (1:500). Horseradish peroxidase tagged secondary antibodies were thereafter applied to the membranes and signals were detected using ECL Prime reagent kit (both GE Healthcare, Uppsala, Sweden). After stripping, membranes were reprobed with a general mouse anti-phospho-tyrosine antibody (1:1000, pY99 #sc7020; Santa Cruz Biotechnology, Dallas, TX, USA) or a mouse anti-phospho-tyrosine-751 PDGFRβ antibody (1:500, pTyr751 #3166; Cell Signaling, Danvers, MA, USA). All membranes were probed with a mouse anti-β-Actin antibody (1:1000, #ab6276; Abcam, Bristol, United Kingdom) as a control for equal loading.

### Intracerebroventricular injections

To perform intracerebroventricular injection of PBS, tPA, or active PDGF-CC, mice were anesthetized with chloral hydrate (450 mg/kg, IP), placed on a stereotactic frame, and injected at bregma −2, mediolateral 0, and dorsoventral 2. Injections contained 3 μl of either PBS, active tPA (3 µM), or active PDGF-CC core protein (3 µM).

### Middle cerebral artery occlusion (MCAO)

Photothrombotic MCAO was induced as described [61, 62]. Briefly, male wild-type, Mac-1^−/−^, *Pdgfra* ^*GFP/*+^, or CX3CR1-GFP/CCR2-RFP mice (10–12 weeks) were anesthetized with chloral hydrate (450 mg/kg Fisher Scientific) alternatively isoflurane and placed securely under a dissecting microscope. The left MCA was exposed and a laser Doppler flow probe (Type N, Transonic Systems) placed on the surface of the cerebral cortex 1.5 mm dorsal median from the bifurcation of MCA. The probe was connected to a flowmeter (Transonic model BLF21) and tissue perfusion units (TPU) recorded with continuous data acquisition (Windaq, DATAQ). A 3.5-mW green laser (540 nm, Melles Griot) was directed at the MCA from a distance of 6 cm, and Rose Bengal (RB) (Fisher) 10 mg/ml in Lactate Ringer’s was injected via the tail vein (50 mg/kg). The TPU was recorded and stable occlusion was achieved when the TPU dropped to less than 20% of pre-occlusion levels and did not rebound within 10 min after laser withdrawal.

### Thrombolysis with tPA

Thrombolysis was carried out as described [61]. Briefly, saline or tPA (10 mg/kg) was administered via a 26G Abbocath^®^-T vascular catheter (Hospira) that was inserted into the tail vein and then connected to a Genie Plus syringe pump (Kent Scientific) via a catheter extension set (Catalog No. IS6003, infusion Devices), trimmed to reduce the priming volume to 200 μl. For tPA thrombolysis, tPA was initiated 5 h after MCAO by slow infusion via the tail vein. All animals were maintained at physiological 37 °C during the infusion process.

### Stroke volume

The assessment of stroke volume was performed essentially as described [61]. Briefly, brains were removed and cut into 2-mm-thick coronal sections and stained with 4% 2,3,5-triphenyltetrazolium chloride (TTC) in PBS for 20 min at 37 °C, and then fixed in 4% paraformaldehyde solution for 10 min. The sections were analyzed with NIH Image J with the following formula [11]:

$$V_{{\% {\text{stroke}}}} = \sum \left( {\text{Areas of lesion}} \right)/\sum \left( {\text{Areas of ipsilateral hemisphere}} \right) \times 100,$$

where *V*_%stroke_ is stroke volume calculated as percent of the ipsilateral hemisphere.

### Intra-cerebral hemorrhage (ICH) volume

A set of digital images was acquired before TTC was developed using the same coronal sections for TTC staining. These images were then analyzed with NIH Image J to quantify ICH volume. All areas with ICH in each image were measured and the total volume of ICH from these images was then integrated using the following formula:

$$V_{\text{ICH}} = \left( {\sum \left( {\text{Areas of ICH}} \right)/2} \right) \times 2,$$

where *V*_ICH_ is ICH volume calculated in cubic millimeter.

### Evans blue dye (EB) extravasation

For analysis of BBB permeability, mice were injected with 100 μl of 4% EB dye (Sigma-Aldrich) intravenously. One hour later, animals were perfused with PBS for 4 min and the brains were removed and separated into hemispheres, and each hemisphere was then homogenized in *N*,*N*-dimethylformamide (Sigma-Aldrich) and centrifuged for 45 min at 25,000 rcf (Eppendorf centrifuge, model 5417R). The supernatants were collected and quantitation of EB extravasation performed as described [69]. For experiments with intracerebroventricular injections data from both hemispheres were combined. For experiments following MCAO, the hemispheres ipsilateral and contralateral to the MCAO were analyzed separately and background EB levels in the non-ischemic hemisphere were subtracted from the ischemic hemisphere. EB levels in each hemisphere were determined from the formula:

$$\left( {A_{{ 6 2 0\;{\text{nm}}}} {-} \, \left( {\left( {A_{{500{\text{nm}}}} + \, A_{{740{\text{nm}}}} } \right)/2} \right)} \right)/{\text{mg wet weight}}.$$

### Epi-fluorescence measurement of BBB permeability

Dextran (tetramethylrhodamine, 70,000 MW, Lysine Fixable) was injected at 8 mg/kg intravenously 23 h after MCAO, and 1 h later, animals were perfused with PBS for 3 min and followed with PFA perfusion for 5 min. The whole brain was then extracted and post-fixed in PFA for 30 min, after which it was switched to PBS immersion. The whole brain is then imaged using Leica MZFL III Stereo/Dissecting microscope and Olympus DP-70 digital camera was used to capture the dorsal view of the entire brain. Epi-fluorescence of the entire dorsal surface was then measured using Image J.

### Analysis of monocyte infiltration after MCAO

Bone-marrow-derived leukocyte infiltration after MCAO was monitored in bitransgenic R/G mice [31, 57]. In the R/G mice, microglia and microglia-derived macrophages are green, monocytes are red, and monocyte-derived macrophages are yellow (expressing both GFP and RFP). To detect the influx of monocytes/macrophages, the following MCAO heterozygous R/G mice were subjected to photothrombosis and euthanized by transcardiac perfusion with PBS following by 4% PFA at 0 (no MCAO), 6, or 24 h after MCAO. The brains were then post-fixed overnight in 4% PFA, transferred to 30% sucrose, and then cryopreserved in OCT prior to preparation of 20 μm frozen sections. To evaluate the distribution of GFP^+^ microglial cells, RFP^+^ monocytes and GFP^+^/RFP^+^ macrophages digital images were captured on a fluorescence microscope and analyzed using Image J (NIH). The number of each cell type in the penumbra was counted and averaged from 3 random fields of 10× objective. There were very few yellow cells apparent at any of the times examined, and therefore, only microglia and monocytes were quantified. The sections were also subjected immunohistochemistry. Briefly, cryosections were permeabilized with 0.25% Tx-100 (PBS) for 10 min, blocked for 1 h at RT in 10% BSA (PBS), incubated with a goat anti-podocalyxin primary antibody (1:200; R&D Systems, UK) overnight at 4 °C, and visualized with a rabbit anti-goat—HRP secondary antibody (1:500; Thermo Fisher Scientific, A16142) in conjunction with the Alexa FluorTM 350 Tyramide Reagent (Thermo Fisher Scientific, B40952). Images (1024 × 1024) were acquired on a Leica SP5X 2-photon confocal microscope and processed using Fiji software.

### Bone-marrow transplantation

Wild-type ROSA or Mac-1^−/−^ ROSA mice, both of which expressed a beta-galactosidase gene, were used as donor and wild-type or Mac-1^−/−^ mice were used as recipient as previously described [30]. In brief, 6-week-old wild-type or Mac-1-deficient mice were irradiated at a dosage of 11 Gy. The lethally irradiated mice were then reconstituted with approximately 4 × 10^6^ wild-type ROSA or Mac-1^−/−^ROSA bone-marrow cells administered intravenously. Mice were allowed to recover for 8–10 weeks prior to MCAO induction.

### Statistics

All values are expressed as mean ± SEM unless otherwise stated. All statistical analysis was performed in GraphPad Prism 7 (GraphPad Software Inc, La Jolla, CA, USA). For data with one independent variable and two groups, a two-tailed unpaired student’s *t* test was used. For analysis of more than two groups with one independent variable, a one-way analysis of variances (ANOVA) was used with Fisher’s least significant difference (LSD) test, and for data with two independent variables, a two-way ANOVA with Fisher’s LSD test was used, and in all cases, *p* values less than 0.05 were considered significant.

## Results

### Primary microglia and the microglial cell line BV2 both enhance the activation of PDGF-CC by tPA

**Fig. 1:**
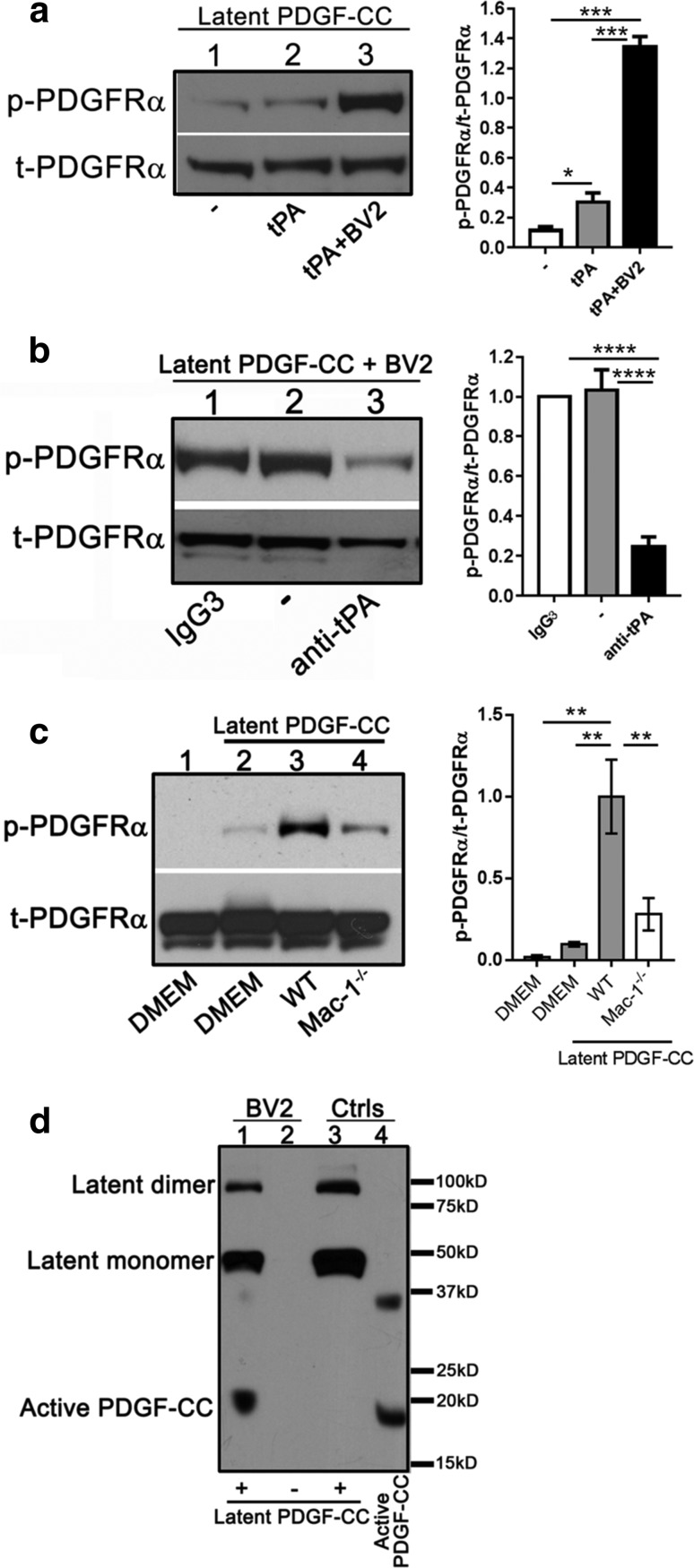
Efficient activation of PDGF-CC by tPA requires microglia. **a** Latent PDGF-CC was incubated with buffer (*lane 1*), 100 nM tPA (*lane 2*), or tPA with BV2 microglial cells (*lane 3*) at 37 °C for 90 min. **b** Latent PDGF-CC was incubated with BV2 cells with the addition of a control IgG3 (*lane 1*), media alone (–) (*lane 2*), or anti-murine-tPA mAb H27B6 (*lane 3*). **c** Primary microglia, prepared from WT and Mac-1^−/−^ neonatal mice, were assessed for their ability to activate latent PDGF-CC as above. *Lane 1*, DMEM control; *lane 2*, latent PDGF-CC; *lane 3*, latent PDGF-CC plus WT microglia; *lane 4*, latent PDGF-CC plus Mac-1^−/−^ microglia. For *panels* **a**–**c**, the amount of active PDGF-CC generated was detected by its ability to stimulate PDGFRα phosphorylation (p-PDGFRα). Total PDGFRα (t-PDGFRα) was used as a loading control. Data shown are the mean ± SD of three independent experiments, *p < 0.05, ***p < 0.001, ****p < 0.0001 (one-way ANOVA with Fisher’s LSD test). **d** BV2 cells were incubated with (*lane 1*) or without (*lane 2*) latent PDGF-CC as above. Generation of active PDGF-CC in the conditioned media was confirmed by immunoblotting with an anti-PDGF-CC antibody. Recombinant latent (*lane 3*) or active (*lane 4*) PDGF-CC was used as controls. Data shown are representative of two independent experiments

**Fig. 2:**
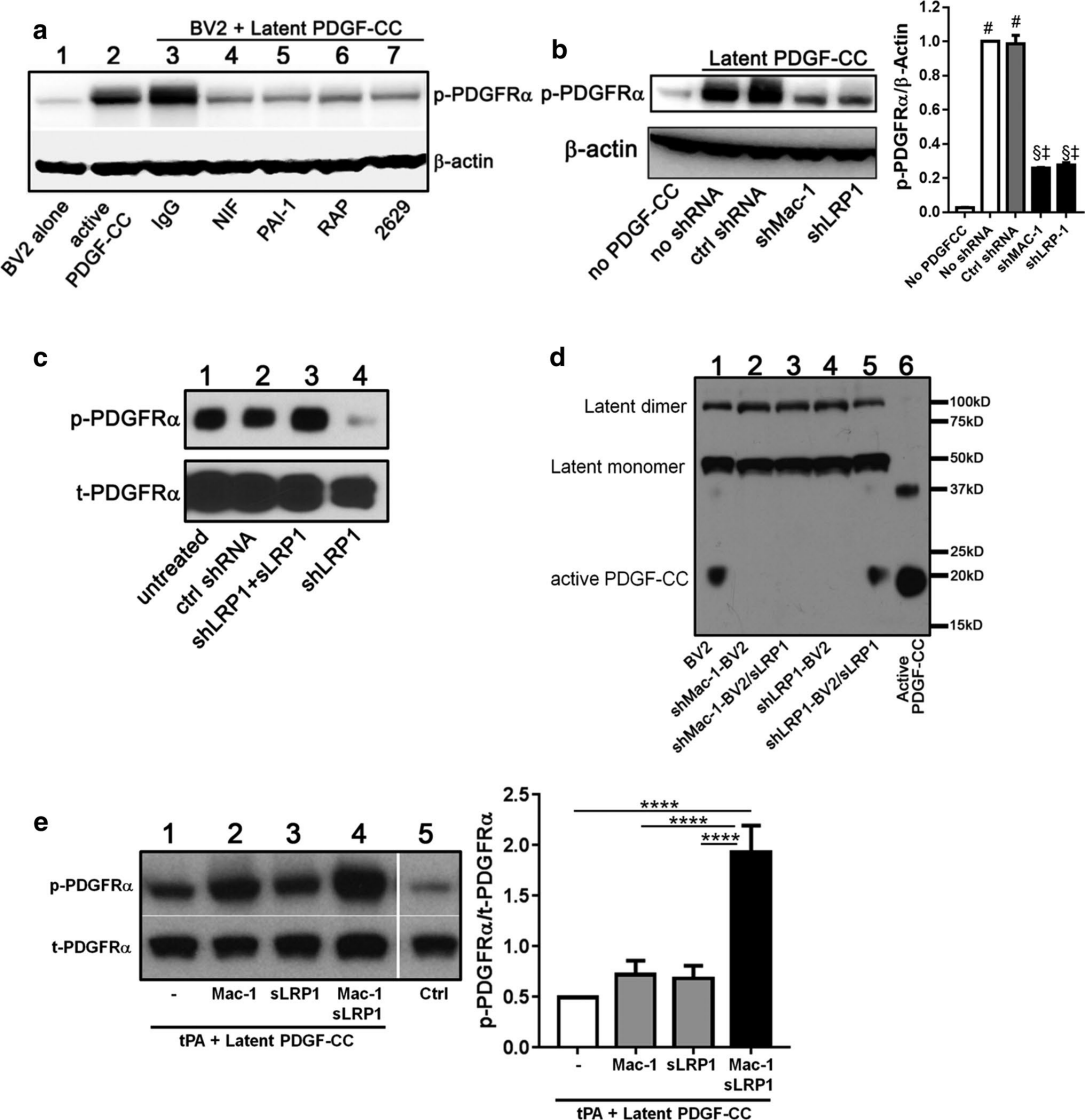
PDGF-CC activation by microglia is dependent on Mac-1 and LRP1. **a** Activation of latent PDGF-CC was performed with BV2 microglial cells in the presence of control IgG (*lane 3*), a Mac-1-specific antagonist NIF (*lane 4*), a tPA inhibitor PAI-1 (*lane 5*), an LRP1 antagonist RAP (*lane 6*), or an LRP1-specific antibody 2629 (*lane 7*). The amount of active PDGF-CC was detected by its ability to stimulate PDGFRα phosphorylation. BV2 alone (*lane 1*) or active PDGF-CC alone (*lane 2*) were used as controls. Data shown are representative of two independent experiments. **b** BV2 cells were infected with lentivirus encoding shRNA for control shRNA (ctrl shRNA), Mac-1 (shMac-1), or LRP1 (shLRP1). The ability of BV2 cells to activate PDGF-CC was assessed as above. Non-infected cells (no shRNA) were used as controls. Data shown are mean ± SD of two independent experiments, ^§^ *p* < 0.001 vs no PDGF-CC, ^#^ *p* < 0.0001 vs no PDGF-CC, ^‡^ *p* < 0.0001 vs no shRNA or control shRNA (one-way ANOVA with Fisher’s LSD test). **c** Latent PDGF-CC was incubated with BV2 (*lane 1*), ctrl shRNA-BV2 (*lane 2*), shLRP1-BV2 plus sLRP1 (*lane 3*), and shLRP1-BV2 (*lane 4*). Generation of active PDGF-CC was determined as above. Data shown are representative of two independent experiments. **d** Latent PDGF-CC was incubated with BV2 (*lane 1*), shMac-1-BV2 (*lane 2*), shMac-1-BV2 plus sLRP1 (*lane 3*), shLRP1-BV2 (*lane 4*), and shLRP1-BV2 plus sLRP1 (*lane 5*). Generation of active PDGF-CC was determined by immunoblotting with an anti-PDGF-CC antibody. Active PDGF-CC (*lane 6*) was used as a control. Data shown are representative of two independent experiments. **e** Latent PDGF-CC was incubated with tPA in the presence or absence of purified Mac-1, sLRP1, or both at 37 °C for 90 min. Active PDGF-CC was detected by its ability to stimulate PDGFRα phosphorylation. *Lane 1*, buffer; *lane 2*, Mac-1; *lane 3*, sLRP1; *lane 4*, Mac-1 and sLRP1; *lane 5*, no latent PDGF-CC (control). Data shown are mean ± SEM of four independent experiments. (****p < 0.0001 one-way ANOVA with Fisher’s LSD test)

Since the activation of PDGF-CC by tPA in vitro is inefficient [19, 61], we examined whether there were co-factors within the central nervous system (CNS) that could promote the activation of PDGF-CC by tPA. For these studies, a cell-based assay was developed, where PDGF-CC stimulated phosphorylation of the PDGFRα was monitored with a cell line (PAE-α) that stably expresses recombinant PDGFRα, but does not express PDGFRβ [16]. Recombinant full-length latent murine PDGF-CC was prepared in insect cells and once activated, its ability to stimulate PDGFRα phosphorylation in PAE-α cells was quantified with a phospho-PDGFRα-specific antibody. Initial experiments demonstrated that consistent with earlier studies [19, 61], the addition of tPA alone to latent PDGF-CC in vitro resulted in very little generation of active PDGF-CC (Fig. 1a, compare lane 1 to lane 2). However, when latent PDGF-CC was incubated with tPA in the presence of the immortalized murine microglial cell line BV2 [28], PDGF-CC activation was significantly enhanced (Fig. 1a, lane 3). Control experiments revealed that conditioned media from the BV2 cells without the addition of latent PDGF-CC does not stimulate PDGFRα activation in PAE-α cells indicating that BV2 cells do not express active PDGF-CC or PDGF-AA which can also signal through the PDGFRα (Fig. 2a, lane 1). These data illustrate that tPA-mediated activation of PDGF-CC is significantly more efficient in the presence of BV2 cells.

Immunoblotting and qRT-PCR experiments indicated that BV2 cells and also primary microglia, which were isolated from wild-type neonatal mice, express endogenous tPA which could support PDGF-CC activation (Online Resource 5). To test this possibility, latent PDGF-CC was incubated with BV2 cells (Fig. 1b) or primary microglia (Fig. 1c), without the addition of exogenous tPA, and these data showed that PDGF-CC could be efficiently activated by BV2 cells alone (Fig. 1b, lane 2) or primary microglia alone (Fig. 1c, lane 3). To confirm that this was due to endogenous tPA expression by BV2 cells, and not due to PDGF-CC cleavage by another protease, a murine-tPA-specific function-blocking antibody, mAb H27B6, was used. This monoclonal antibody specifically inhibits the enzymatic activity of tPA, and was able to neutralize BV2-mediated PDGF-CC activation (Fig. 1b, lane 3), while the addition of a control IgG_3_ had no effect on activation (Fig. 1b, lane 1). tPA is a physiological activator of plasminogen, which generates an active enzyme plasmin. To investigate whether activation of PDGF-CC by tPA is mediated indirectly by plasmin, a potent plasmin inhibitor, aprotinin, was added in the activation assay. These results showed that the addition of aprotinin failed to block PDGF-CC activation (Online Resource 6), indicating that plasmin is not required for activation of PDGF-CC by tPA.

To confirm that the generation of active PDGF-CC resulted from its proteolytic cleavage by tPA, conditioned media from the BV2 cells with and without added latent PDGF-CC were collected and subjected to SDS-PAGE followed by immunoblotting with anti-PDGF-CC antibody. These data demonstrated that incubation of latent PDGF-CC with BV2 cells resulted in the generation of the 20 kDa active PDGF-CC core domain (Fig. 1d, lane 1) and that the BV2 cells do not express endogenous PDGF-CC (Fig. 1d, lane 2). Together, these data indicate that BV2 cells and primary microglia can promote the activation of PDGF-CC by tPA and suggest that microglia may express co-factors that enhance the interaction between tPA and latent PDGF-CC.

### Activation of PDGF-CC by microglia requires Mac-1 and LRP1

Microglia express both Mac-1 [46] and LRP1 [7] (Online Resource 2). Previously, we reported that LRP1 is necessary for tPA to increase BBB permeability [51, 61, 69] and that Mac-1 interacts with both tPA and LRP1, forming a ternary complex [9]. Therefore, we examined whether LRP1, Mac-1, or both, could promote efficient PDGF-CC activation by tPA. For these studies, latent PDGF-CC was incubated with BV2 cells in the presence or absence of specific antagonists of Mac-1, LRP1, or tPA. Neutrophil inhibitory factor (NIF) is an antagonist of Mac-1 [48], and the addition of NIF to BV2 cells resulted in a significant reduction in phosphorylated PDGFRα, indicating that blocking Mac-1 ligand binding by NIF reduces PDGF-CC activation (Fig. 2a, lane 4). Similarly, PDGF-CC activation by BV2 cells could also be blocked using an LRP1 antagonist, receptor-associated protein (RAP) (Fig. 2a, lane 6). However, since RAP not only inhibits LRP1, but also blocks ligand binding to other LDL receptor family members [6, 8, 36], we also tested an LRP1-specific neutralizing antibody (2629) and found it to block PDGF-CC activation comparable to RAP (Fig. 2a, lane 7). Finally, cells were treated with the physiological inhibitor of tPA, plasminogen activator inhibitor 1 (PAI-1), and similar to the effects of the anti-tPA antibody treatment, PAI-1 inhibited PDGF-CC activation (Fig. 2a, lane 5).

To confirm that the antagonists used were specifically blocking Mac-1 and LRP1 on BV2 cells and not acting through off-target interactions, the expression of Mac-1 and LRP1 in BV2 cells was silenced by RNA interference using lentiviral constructs expressing small hairpin RNAs (shRNA) specific for Mac-1 or LRP1. These shRNAs markedly inhibited the expression of Mac-1 and LRP1 (Online Resource 3). When latent PDGF-CC was incubated with either of these shRNA-knock-down BV2 cells, the activation of PDGF-CC was decreased by over 70% in both knock-down cell lines, compared to control shRNA (ctrl shRNA) cells (Fig. 2b and Online Resource 7). Moreover, we found that the addition of sLRP1 to LRP1-knock-down BV2 cells rescued their ability to activate PDGF-CC and induced PDGFRα phosphorylation (Fig. 2c, compare lanes 3 and 4, and Fig. 2d, compare lanes 4 and 5). Finally, we found that unlike the results with LRP1-knock-down BV2 cells, sLRP1 was unable to rescue the ability of Mac-1-knock-down BV2 cells to promote PDGF-CC cleavage (Fig. 2d, compare lanes 3 and 5), confirming that both Mac-1 and LRP1 are required for efficient activation of PDGF-CC. Collectively, these data provide strong support for the hypothesis that Mac-1 and LRP1 are co-factors for the activation of PDGF-CC by tPA. To test whether Mac-1 and LRP1 are sufficient to promote efficient PDGF-CC activation by tPA, or if other cellular factors were also required, we developed a cell-free activation assay using purified Mac-1, sLRP1, tPA, and PDGF-CC. As shown in Fig. 2e, mixing tPA with either Mac-1 or sLRP1 alone failed to support efficient PDGF-CC activation. In contrast, the addition of both Mac-1 and sLRP1 with tPA led to significantly more efficient activation of PDGF-CC. Together, these results demonstrate that both Mac-1 and LRP1 are necessary and sufficient to facilitate the efficient activation of PDGF-CC by tPA.

### LRP1, Mac-1, and PDGFRα-expressing cells are all located within the NVU

**Fig. 3:**
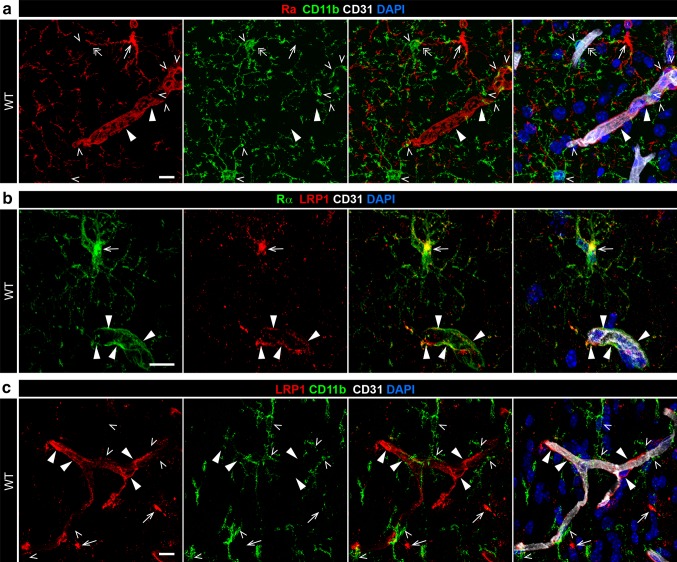
CD11b, LRP1, and PDGFRα are expressed in the NVU of wild-type (WT) mice. 50 μm vibratome sections from WT murine brains were analyzed by immunofluorescence staining and confocal microscopy for CD11b (the alpha chain of Mac-1), LRP1, PDGFRα, and the endothelial cell marker CD31 (*white*) to visualize vessels. **a** PDGFRα (*red*) is expressed both in nonvascular cells (*arrows*) and in cells associated with a subset of cerebral vessels, presumably arterioles (*closed arrowheads*), but not in capillaries (*double arrows*). CD11b (*green*) is seen throughout the field of view in resting microglia with their characteristic ramified appearance with occasional branching processes contacting both PDGFRα positive vessels, as well as PDGFRα negative microvasculature (*open arrowheads*). **b** LRP1 (*red*) and PDGFRα (*green*) are co-localized in both nonvascular cells (*arrows*) and vessel-associated cells (*closed arrowheads*). **c** CD11b (*green*) is not co-expressed by LRP1 positive cells (*red*), neither by nonvascular (*arrows*) or by vessel-associated LRP1 positive cells (*closed arrowheads*). However, several CD11b branching processes are contacting LRP1 positive vessels (*open arrowheads*). The images were captured in the cerebral cortex (**a**, **b**) and in the hippocampus (**c**) and display the maximum intensity projections generated from confocal Z-stacks. *Scale bars* 10 μm

Previously, we demonstrated that tPA, PDGF-CC, and the PDGFRα were all localized to the NVU [20, 21, 51, 61], and the data above showing the requirement of LRP1 and Mac-1 for efficient activation of PDGF-CC imply that they too should be expressed in the NVU. Therefore, we investigated if both Mac-1 and LRP1 were associated with the NVU in proximity to PDGFRα-expressing cells. For these studies, sections from wild-type murine brains were analyzed by immunofluorescence staining and confocal microscopy for CD11b (the alpha chain of Mac-1), LRP1, PDGFRα, and the endothelial cell marker CD31 to visualize vessels. These data demonstrated that all three proteins, Mac-1, LRP1, and PDGFRα, are located in close proximity to each other on the abluminal side of the NVU (Fig. 3). Panel a shows PDGFRα (red) both in nonvascular cells (arrows) and in cells associated with a subset of cerebral vessels, presumably arterioles (closed arrowheads), but not in capillaries (double arrows). CD11b (green) is seen throughout the field of view in resting microglia with their characteristic ramified appearance and with occasional branching processes contacting both PDGFRα positive vessels, as well as PDGFRα negative microvasculature (open arrowheads). Panel b demonstrates prominent overlap between LRP1 (red) and PDGFRα (green) in both nonvascular cells (arrows) and in vessel-associated cells (closed arrowheads). Finally, panel c shows that CD11b (green) is not expressed by LRP1 positive cells (red), neither by nonvascular (arrows) or by vessel-associated LRP1 positive cells (closed arrowheads). However, several CD11b branching processes are contacting LRP1 positive vessels (open arrowheads). These data, together with the previously published localization of tPA, PDGF-CC, and PDGFRα to arterioles in the brain [20, 21, 61], support the hypothesis that Mac-1 and LRP1 could promote signaling by the PDGFRα in the NVU by acting as co-factors for tPA-mediated activation of PDGF-CC.

### Mac-1-deficiency prevents tPA- but not active PDGF-CC-induced BBB opening

**Fig. 4:**
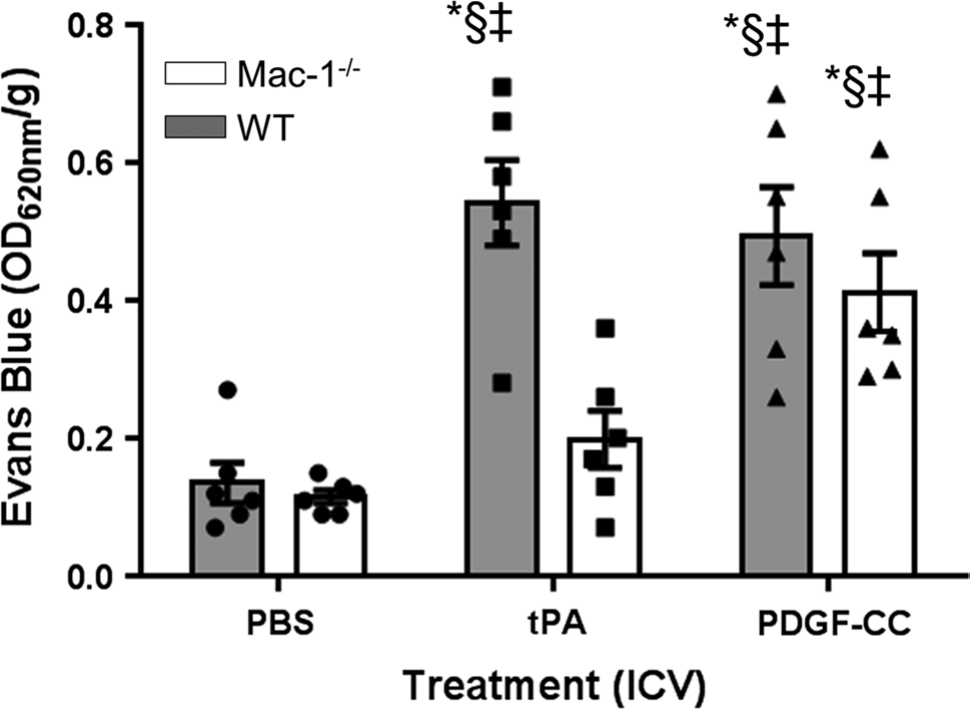
Mac-1 deficiency prevents tPA-mediated BBB permeability, but responds to active PDGF-CC. Wild-type and Mac-1^−/−^ mice were anesthetized and injected with PBS, tPA, or active PDGF-CC intracerebroventricularly. One hour later, EB dye was injected intravenously and animals were perfused with PBS 1 h later. The brains were then harvested and EB extravasation quantified. The *symbols* indicate each individual data point with the *bars* representing the mean values ± SEM. The significance for all comparisons of each condition in each genotype was determined from a two-way ANOVA with a Fisher’s LSD test (Online Resource 9); *p < 0.001 vs WT injected with PBS, ^§^ *p* < 0.001 vs Mac-1^−/−^ injected with PBS, and ^‡^ *p* < 0.01 vs Mac-1^−/−^ injected with tPA. In each group, *n* = 6

Following ischemic stroke, there is a significant tPA-dependent increase in cerebrovascular permeability via signaling through the PDGF-CC/PDGFRα pathway [61, 69]. In addition, in the absence of an ischemic insult, the intraventricular injection of tPA into the cerebrospinal fluid (CSF) of healthy mice can directly increase the cerebrovascular permeability via signaling through the PDGF-CC/PDGFRα pathway [61, 69]. Thus, tPA-dependent activation of PDGF-CC within the NVU is sufficient to induce BBB permeability. To determine if tPA-induced BBB permeability is dependent on Mac-1, we injected tPA or active PDGF-CC intraventricularly into wild-type or Mac-1^−/−^ mice [42] under non-ischemic conditions and examined vascular permeability using EB dye extravasation. These data demonstrated that consistent with previous studies [61, 69], the intraventricular injection of tPA into wild-type mice induced a significant increase in BBB permeability within 2 h (Fig. 4); however, in contrast to wild-type mice, the intraventricular injection of tPA into Mac-1^−/−^ mice did not increase permeability of the BBB (Fig. 4). This suggests that Mac-1 is required for the in vivo effects of tPA on the BBB. To determine if Mac-1 was also required for the PDGFRα-mediated effect on BBB permeability that is induced by active PDGF-CC, BBB permeability following intraventricular administration of active PDGF-CC was examined in both wild-type and Mac-1^−/−^ mice. These data demonstrated that unlike tPA, active PDGF-CC can induce BBB permeability in both wild-type and Mac-1^−/−^ mice (Fig. 4). This suggests that the activation of endogenous latent PDGF-CC by tPA in vivo requires Mac-1, but that once PDGF-CC is active, it acts downstream of Mac-1. These data support the hypothesis that Mac-1 is a cofactor for tPA-mediated activation of PDGF-CC within the NVU.

### Mac-1 deficiency preserves BBB integrity after MCAO

**Fig. 5:**
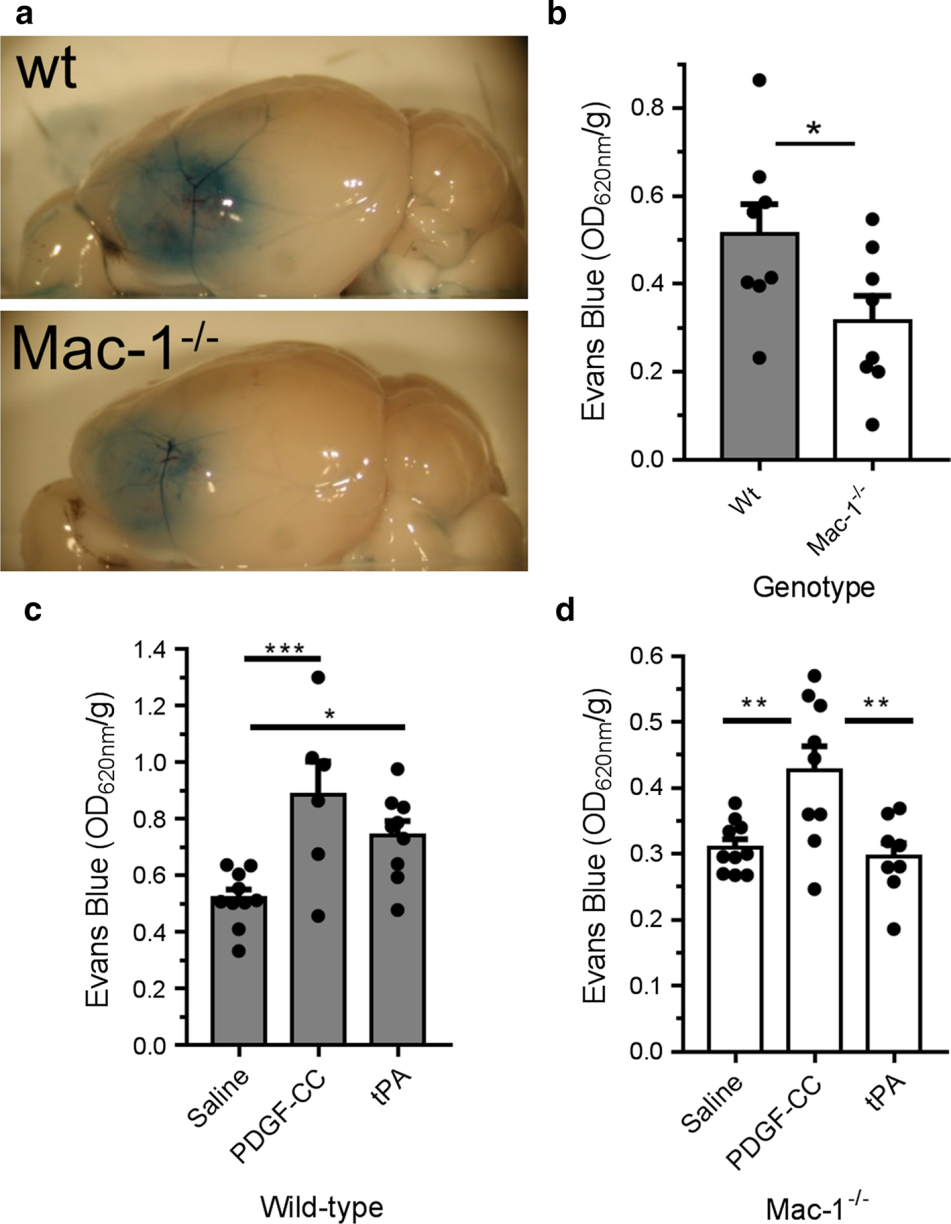
Active PDGF-CC but not tPA restores BBB permeability after MCAO in Mac-1-deficient mice. Wild-type (WT) and Mac-1^−/−^ mice were subjected to MCAO, and 23 h later, EB dye was injected intravenously as a bolus and animals were perfused 1 h later with PBS. **a** Representative images of the ipsilateral hemisphere 24 h after MCAO from WT and Mac-1^−/−^ mice. **b** Quantification of the EB extravasation 24 h after MCAO. WT and Mac-1^−/−^ mice were subjected to MCAO, and 1 h later, the mice were subjected to intracerebroventricular (ICV) injection with saline, active PDGF-CC, or tPA. Twenty-three hours later, EB dye was injected intravenously as a bolus and animals were perfused 1 h later with PBS. **c**, **d** WT and Mac-1^−^/^−^ mice were subjected to MCAO followed 1 h later by intracerebroventricular (ICV) injection with saline, active PDGF-CC or tPA; 23 h after MCAO, EB dye was injected intravenously and the mice were perfused with PBS 24 h after MCAO. Quantification of EB extravasation in WT (**c**) and Mac-1^−/−^ (**d**) mice. In each group, *n* = 8–10; errors represent SEM; *p < 0.05; **p < 0.01 (one-way ANOVA with Fisher’s LSD test)

We next examined the effect of Mac-1 deficiency on BBB permeability after MCAO in wild-type and Mac-1^−/−^ mice using EB dye (Fig. 5). The results of this analysis indicated that in the ischemic hemisphere, there was a 40% reduction in EB extravasation in Mac-1^−/−^ mice compared to wild-type mice 24 h after MCAO (Fig. 5a, b). To investigate if this effect was due to Mac-1 acting as a co-factor for the activation of latent PDGF-CC, recombinant active PDGF-CC or tPA, or saline was injected directly to the CSF of wild-type (Fig. 5c) or Mac-1^−/−^ (Fig. 5d) mice 1 h after MCAO induction. Then, 24 h after MCAO, BBB permeability was analyzed by EB dye extravasation. These data confirmed that active PDGF-CC but not tPA was able to increase BBB permeability in Mac-1^−/−^ mice, whereas both PDGF-CC and tPA are able to increase BBB permeability in wild-type mice (Fig. 5c, d). This demonstrates that the protection of the BBB noted in Mac-1^−/−^ mice following MCAO can be largely abolished by the addition of active PDGF-CC and further supports the hypothesis that active PDGF-CC is downstream of Mac-1 and that tPA requires Mac-1 to induce BBB permeability after MCAO.

### Ischemic stroke induces microglial activation

**Fig. 6:**
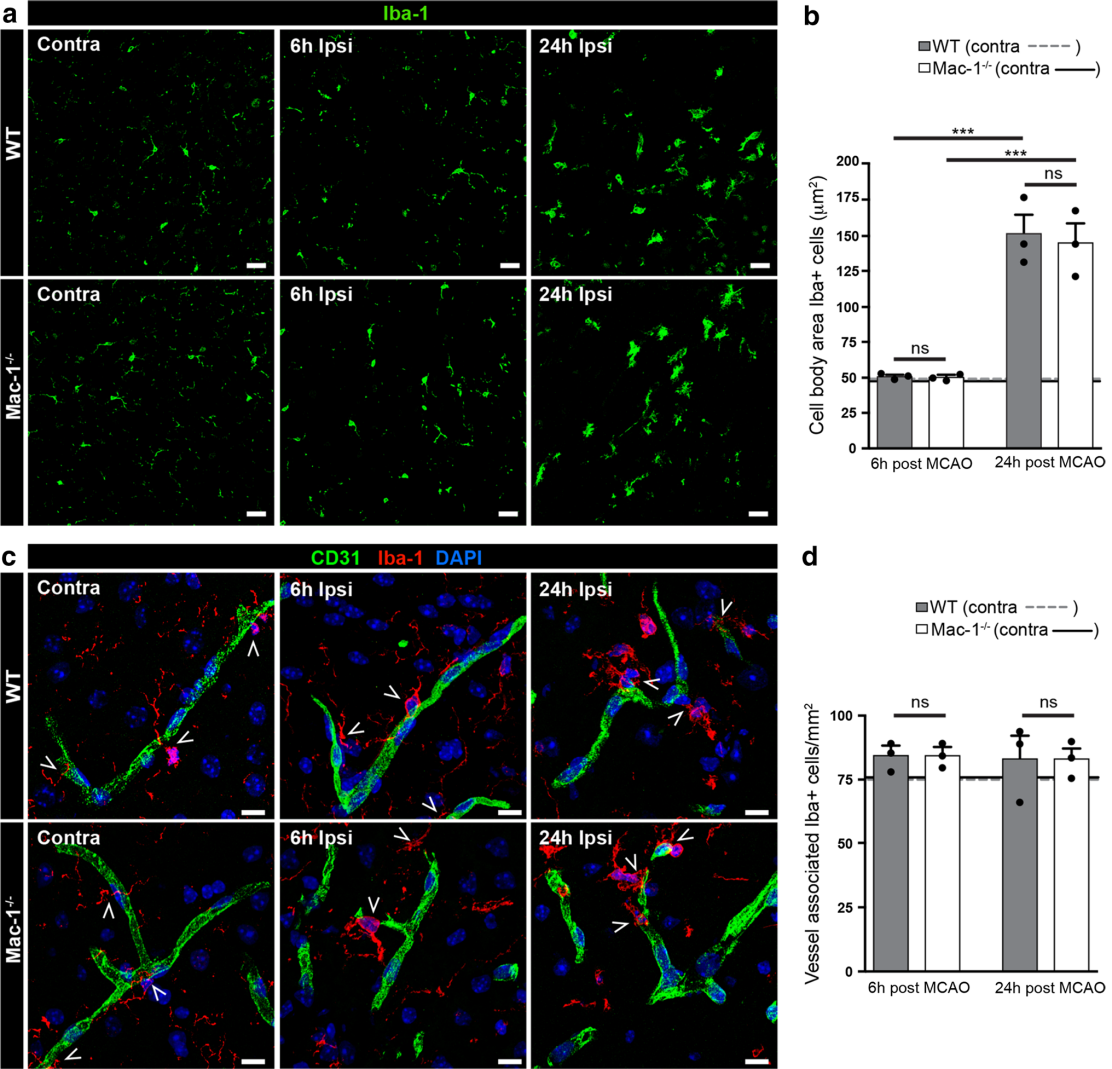
Ischemic stroke induces microglial activation. Immunofluorescent staining for the microglial/macrophage marker Iba-1 was performed on 12 μm brain sections from wild-type (WT) and Mac-1^−/−^ mice 6 and 24 h after MCAO (*n* = 3 per genotype and timepoint). **a** Iba-1 expressing cells (*green*) in the ischemic border have largely adopted an amoeboid morphology 24 h after MCAO in both WT and Mac-1^−/−^ mice. **b** Five confocal images per brain were evaluated using Volocity. **c** Iba-1 positive cells (*red*) are associated with cerebral vessels (visualized with CD31, *green*) in both the non-ischemic hemisphere and the ischemic penumbra 6 and 24 h after MCAO (*open arrowheads*). **d** Five confocal images per brain were evaluated using Image J. The images were captured in the cerebral cortex. Cell nuclei were visualized by DAPI (*blue*). Data are presented as the group mean values ± SEM. Statistical significance was determined by one-way ANOVA with Fisher’s LSD test and ***P < 0.001; *ns* non-significant. *Scale bars* **a** 25 μm, **c** 10 μm

Microglial activation is reported to occur early following ischemic stroke [45] and include increased expression of Mac-1 as early as 6 h after MCAO [23, 50]. We, therefore, investigated whether Mac-1 deficiency affected the activation of resident brain microglia following MCAO. For this analysis, immunofluorescent staining for the microglial/macrophage marker Iba-1 was performed followed by image analysis for a quiescent (ramified) or reactive (amoeboid) morphology. These data demonstrated that in both wild-type and Mac-1^−/−^ mice, Iba-1 expressing cells in the ischemic border begin to lose their ramified appearance as early as 6 h after MCAO and that by 24 h Iba-1 positive cells in both genotypes have largely adopted an amoeboid morphology indicative of reactive microglia (Fig. 6a, b). This suggests that Mac-1 ablation does not inhibit microglial activation after MCAO. We also examined whether Mac-1 deficiency affected the association of Iba-1 positive cells with cerebral vessels in both the non-ischemic hemisphere and the ischemic penumbra 6 and 24 h after MCAO. This analysis indicated that there was no difference in the association of Iba-1 expressing cells with the NVU in wild-type or Mac-1^−/−^ mice and that the extent of this association in the ischemic penumbra did not change following MCAO (Fig. 6c, d). Together, these data show that during ischemic stroke, microglial activation in the ischemic border is Mac-1 independent and that Mac-1 deficiency does not affect microglial interaction with the NVU.

### Mac-1 deficiency attenuates PDGFRα activation after MCAO

**Fig. 7:**
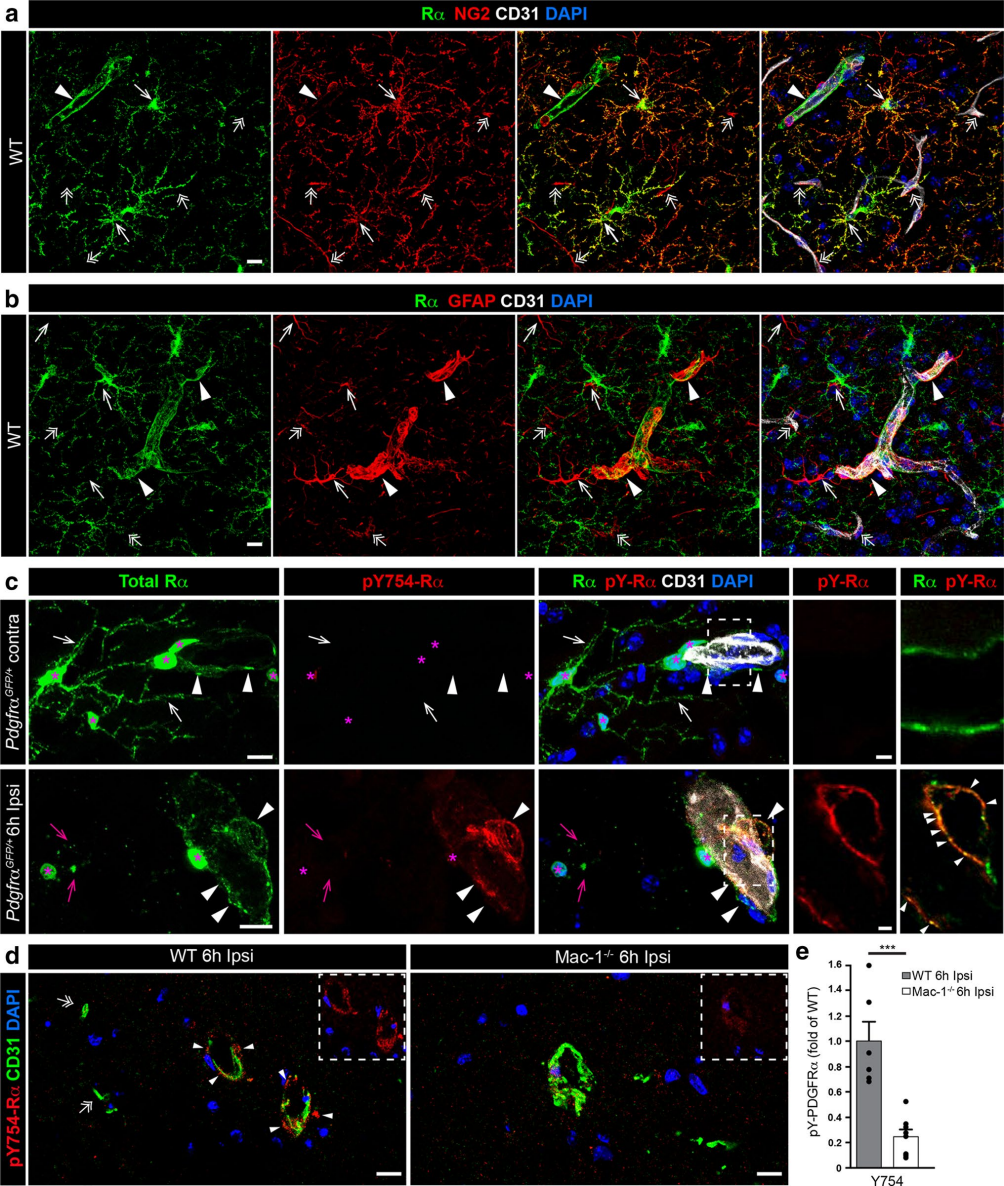
Mac-1 deficiency attenuates PDGFRα activation after MCAO. **a** In healthy wild-type (WT) brains, PDGFRα (*green*) is expressed in nonvascular NG2 (*red*) positive glia cells (*arrows*), as well as in perivascular cells localized around a subpopulation of vessels, presumably arterioles (*closed arrowheads*). NG2 positive pericytes in capillaries were consistently PDGFRα negative (*double arrows*). Vessels were visualized with CD31 (*white*). **b** Subpopulation of perivascular GFAP-positive astrocytes co-expresses PDGFRα (*closed arrowheads*), whereas nonvascular GFAP-positive astrocytes (*arrows*) or GFAP-positive cells around capillaries (*double arrows*) were PDGFRα negative. **c** Immunofluorescent staining using antibodies that specifically recognize activated PDGFRα (pY-754, *red*) in murine brain sections from *Pdgfrα* ^*GFP/*+^ mice demonstrates robust phosphorylation of PDGFRα (*arrowheads*) in the infarct border (ipsi) 6 h after MCAO, specifically around a subset of cerebral vessels (CD31, *white*). No apparent PDGFRα phosphorylation was observed in the cell population that was not associated with vessels or in any cells in the contralateral non-ischemic hemisphere. Note the co-localization of GFP positive nuclei (*magenta asterisks*) with immunofluorescence for PDGFRα protein (*green*) in both nonvascular (*arrows*) and perivascular (*closed arrowheads*) cells in the contralateral hemisphere, thus confirming antibody specificity. **d** For quantification, unfixed brains from Mac-1 deficient (*n* = 8) and WT (*n* = 6) mice subjected to 6 h of MCAO were stained with the phospho-specific PDGFRα antibodies (*red*) and CD31 (*green*). **e** Four-to-seven epifluorescent images per brain were evaluated using Image J and the area of antibody immunoreactivity was determined and expressed as fold of WT. The analysis shows that PDGFRα phosphorylation was significantly reduced in Mac-1^−/−^ mice compared to WT mice. Data presented the group mean values ± SEM. Statistical evaluation was performed using unpaired *t* test and ***p < 0.001. Cell nuclei were visualized by DAPI (*blue*). The pictures are representative images from the respective staining and were captured in the penumbra of the ipsilateral side. The images display the maximum intensity projections generated from confocal Z-stacks. *Scale bars* 10 μm (**a**–**d**) and 2 μm (**c**, *inserts*)

To determine if Mac-1 deficiency directly affects PDGFRα activation during ischemic stroke, we first examined the localization of the PDGFRα by confocal microscopy. These data indicate that as previously reported, in healthy wild-type mice, there is prominent expression of PDGFRα in at least two different cell types, nonvascular NG2 positive glia cells (Fig. 7a, arrows) [55], and a subset of GFAP-positive perivascular astrocytes associated with arterioles [18, 21, 61] (Fig. 7b, closed arrowheads). PDGFRα expression was not detected in NG2 positive pericytes around capillaries (Fig. 7a, double arrows), in nonvascular GFAP-positive astrocytes (Fig. 7b, arrows) or GFAP-positive cells around capillaries (Fig. 7b, double arrows). Next PDGFRα activation after MCAO was examined in *Pdgfrα* ^*GFP/*+^ reporter mice [25]. Confocal analysis showed co-localization of GFP positive nuclei (magenta asterisks) with immunofluorescence for PDGFRα protein (green) in both nonvascular (Fig. 7c, arrows) and perivascular (Fig. 7c, closed arrowheads) cells in the contralateral hemisphere, thus confirming antibody specificity. At 6 h after MCAO, PDGFRα was activated specifically around arterioles in the ischemic penumbra as visualized using phospho-tyrosine specific PDGFRα antibodies (pY754 and pY1018 [15, 56]) (lower panel Fig. 7c, closed arrowheads and Online Resource 8). In contrast, no apparent PDGFRα phosphorylation was observed in the cell population that was not associated with vessels, or in any cells in the contralateral non-ischemic hemisphere. It was also noted that the total PDGFRα expression in the non-vessel-associated cells was reduced in the ischemic penumbra, although the GFP nuclear signal remained in these cells (Fig. 7c and Online Resource 8). Finally, quantitative analysis of PDGFRα phosphorylation was performed in wild-type and Mac-1^−/−^ mice by image analysis with the two different PDGFRα specific phospho-tyrosine antibodies pY754 (Fig. 7d, e) and pY1018 (Online Resource 8c). These data indicated that in wild-type mice 6 h after MCAO, there was robust phosphorylation of PDGFRα around cerebral arterioles (Fig. 7d, closed arrowheads) but not capillaries (double arrows) in the infarct border, whereas in Mac-1^−/−^ mice, PDGFRα phosphorylation was significantly decreased.

### Leukocyte infiltration after MCAO

**Fig. 8:**
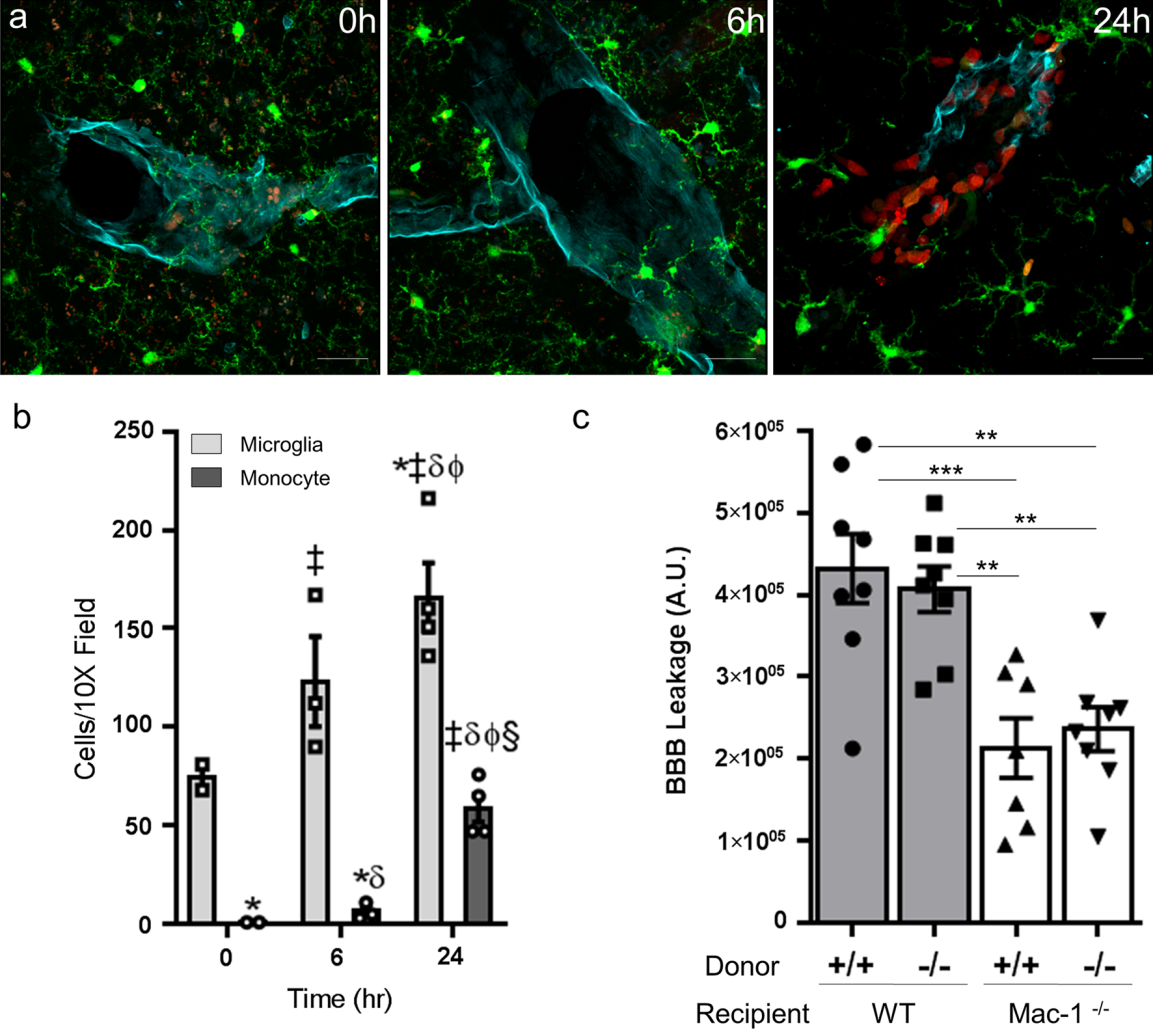
Time course of monocytes infiltration after MCAO in R/G mice. **a** R/G mice subjected to MCAO showed a clear increase in RFP^+^ monocyte infiltration overtime. Representative time course images of the ischemic penumbra from RFP^+^ (monocytes/macrophages) and GFP^+^ (microglia) brain sections stained for the endothelial cell marker podocalyxin (*cyan*). At 0 (no MCAO) and 6 h post-MCAO, there are few detectable RFP^+^ monocytes infiltrating the parenchyma near blood vessels in the ischemic penumbra. By 24 h, a significant number of RFP^+^ monocytes can be observed not only in the blood vessels, but also in the parenchyma (*scale bars* 25 μm). **b** Quantification of GFP^+^ and RFP^+^ cells (*n* = 2–4; errors represent SEM). The significance for all comparisons was determined from a two-way ANOVA with a Fisher’s LSD test (Online Resource 10); *p < 0.05 vs 0 h microglia, ^‡^ *p* < 0.05 vs 0 h monocyte, ^δ^ *p* < 0.05 vs 6 h microglia, ^ϕ^ *p* < 0.05 vs 6 h monocyte, ^§^ *p* < 0.0001 vs 24 h microglia. **c** Mac-1-mediated BBB permeability is independent of infiltrating leukocytes. Wild-type (WT) and Mac-1^−/−^ mice received bone marrow transplant from either WT or Mac-1^−/−^ mice, and 8–10 weeks later, they were subjected to MCAO. BBB permeability was quantified by fluorescent microscopy and image analysis of 70-kDa rhodamine-dextran extravasation 24 h after MCAO. In each group, *n* = 7–8; errors represent SEM, **p < 0.01, ***p < 0.001 (two-way ANOVA with Fisher’s LSD test)

To determine whether Mac-1 expressed on infiltrating leukocytes or on resident microglia/macrophages (or both) contributed to PDGF-CC activation and subsequent BBB dysfunction following MCAO, we utilized the CX3CR1-GFP/CCR2-RFP (R/G) mice. These mice express GFP in microglia and macrophages under the control of the CX3CR1 promoter and RFP in monocytes and macrophages under the control of the CCR2 promoter. This bitransgenic system offers a simple way to distinguish microglia (green) from monocytes (red) and monocyte-derived macrophages (yellow, both GFP and RFP), and is commonly used to assess neuroinflammation after injury [4, 31, 57]. We subjected these heterozygous R/G mice to MCAO, and examined the recruitment of microglia and the influx of macrophage/monocytes into the ischemic penumbra following MCAO at 0, 6 and 24 h after MCAO (Fig. 8a). Representative confocal images are shown in Fig. 8a and they demonstrated that prior to MCAO (0 h) and at 6 h after MCAO GFP^+^ microglia are the dominant cell-type present, whereas by 24 h after MCAO, there are many RFP^+^ monocytes both associated with cerebral vessels and infiltrated into the brain parenchyma. Quantification of the different cell types indicates that the infiltration of peripheral leukocytes is not significant at 6 h after MCAO, but is highly significant by 24 h after MCAO (Fig. 8b). These data indicate that resident CNS microglial cells are the likely source of Mac-1 that drive the early activation of the PDGFRα noted at 6 h after MCAO (Fig. 7 and Online Resource 8).

To confirm that CNS resident Mac-1 expressing cells and not infiltrating leukocytes are promoting the early loss of BBB integrity bone marrow transplantation experiments were performed. For these studies, Mac-1^−/−^ or wild-type (WT) bone marrow was transplanted into lethally irradiated WT or Mac-1^−/−^ mice. The WT → Mac-1^−/−^ mice express Mac-1 in bone-marrow-derived cells, but lack Mac-1 in resident microglia, whereas the Mac-1^−/−^ → WT mice will lack Mac-1 in bone-marrow-derived leukocytes, but express Mac-1 in resident microglia. As controls for the effects of radiation and transplantation both Mac-1^−/−^ → Mac-1^−/−^ and WT → WT mice were also examined. Full bone-marrow reconstitution was achieved by approximately 10 weeks after myeloablation. These animals were then subjected to MCAO followed by intravenous injection of 70-kDa rhodamine dextran 23 h later. The mice were then extensively perfused at 24 h after MCAO to allow for quantitative analysis of the extent of BBB permeability by fluorescent microscopy. These data demonstrated that wild-type bone-marrow transplantation into Mac-1^−/−^ mice did not restore BBB permeability to wild-type levels. Likewise, transplantation of Mac-1^−/−^ marrow into WT mice did not provide protection of the BBB. Finally, transplantation of WT bone marrow into WT mice or Mac-1^−/−^ bone marrow into Mac-1^−/−^ mice did not alter the phenotype of either genotype indicating that the effects on BBB permeability were not due to the radiation or transplantation (Fig. 8c). These results are consistent with the observations that there are very few infiltrating leukocytes in the first 6 h after MCAO, and they indicate that the protection to the BBB seen in the first 24 h after MCAO in Mac-1^−/−^ mice is independent of Mac-1 on circulating leukocytes and instead is dependent upon Mac-1 expression in resident microglia.

### Mac-1 deficiency protects against tPA-induced ICH after MCAO

**Fig. 9:**
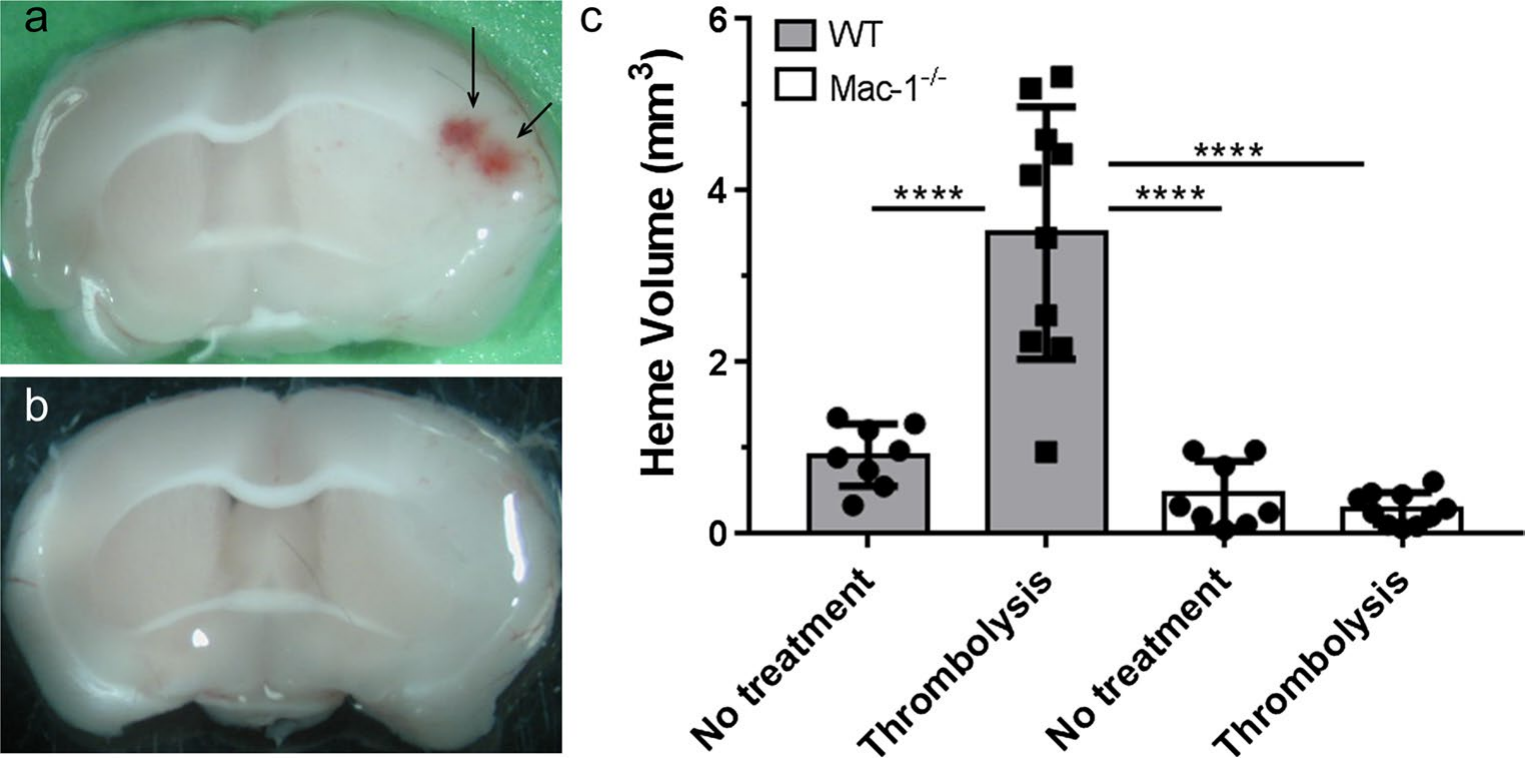
Intra-cerebral hemorrhage (ICH) associated with tPA-induced thrombolysis after MCAO was attenuated in Mac-1^−/−^ mice. Wild-type (WT) and Mac-1^−/−^ mice received a late thrombolysis with tPA 5 h after MCAO. **a** Representative images of ICH in WT and Mac-1^−/−^ mice 72 h after MCAO. **b** Quantification of ICH volume. In each group, *n* = 8–10; errors represent SEM, ****p < 0.0001 (two-way ANOVA with Fisher’s LSD test)

The data above indicate that Mac-1 plays a critical role in tPA-mediated PDGF-CC activation and signaling through the PDGFRα leading to increased BBB permeability. This suggests that by promoting tPA-mediated PDGF-CC activation Mac-1 may play a role in the development of spontaneous ICH associated with thrombolytic tPA treatment for ischemic stroke. Therefore, we examined whether Mac-1 deficiency could reduce the risk of ICH associated with late tPA-mediated thrombolysis initiated 5 h after the induction of MCAO. For these studies, thrombolytic tPA was administered to wild-type and Mac-1^−/−^ mice by intravenous injection 5 h after MCAO, and 3 days later, the mice were euthanized and spontaneous ICH was determined by image analysis. These data show that in wild-type mice, there is a significant increase in spontaneous ICH with late tPA treatment, but that in Mac-1^−/−^ mice, no such increase is observed (Fig. 9). This suggests that the known association of thrombolytic tPA with hemorrhagic complications may be due in part to Mac-1-dependent tPA-mediated signaling through the PDGFRα.

## Discussion

The unique functions of CNS require a tightly regulated extracellular environment that is maintained by the BBB [26, 49, 59]. In the healthy brain, the NVU controls the dynamics of the BBB facilitating the two-way exchange between the CNS and blood necessary for the maintenance of brain homeostasis. Under pathologic conditions such as cerebral ischemia, this tight control is rapidly compromised; however, the early loss of barrier integrity is transient and there is a period of recovery, which is followed by a second phase of increased BBB permeability [35, 37]. This early rise and fall in BBB permeability demonstrates that the BBB is not just a simple physical barrier; rather, the movement of protein and non-protein molecules through the BBB is a regulated process. Consistent with this, our previous results have shown that proteolytic activation of PDGF-CC by tPA in the NVU can rapidly increase BBB permeability in both the healthy and pathologic brain via signaling through the PDGFRα [21, 61]. Given the importance of maintaining the extracellular environment of the brain while also being responsive to physiologic changes in the blood, then any process that is able to compromise the function of the BBB must be very tightly regulated. In the investigation presented here, we identify a novel mechanism that controls the BBB by regulating PDGFRα signaling in the NVU. This is achieved by restricting the activation of PDGF-CC by tPA to sites, where Mac-1 and LRP1 are enriched, such as in the NVU. We demonstrated in vitro with purified proteins and in cell culture studies that both Mac-1 and LRP1 are required for efficient activation of PDGF-CC by tPA and that antagonism of either Mac-1 or LRP1 either by specific protein ligands such as NIF or RAP, or by shRNA, or by genetic deletion significantly reduced PDGF-CC cleavage and activation. In addition, we demonstrated that this pathway is functional in vivo by showing that in healthy mice, genetic deficiency of Mac-1 protected against increased BBB permeability induced by intraventricular injection of tPA into the CSF but not from injection of active PDGF-CC. Taken together with our previous studies showing that antagonism of either tPA or LRP1 in vivo similarly protects against tPA-mediated increased BBB permeability [69] but not against permeability induced by intraventricular injection of active PDGF-CC [61], these results establish for the first time that Mac-1 and LRP1 are co-factors regulating tPA activation of PDGF-CC in the NVU.

Consistent with the cleavage and functional data, and with the importance of regulating PDGFRα signaling, confocal immunofluorescence analysis of the NVU revealed localization of both LRP1 and Mac-1 to PDGFRα positive arterioles in the uninjured brain. However, in response to cerebral ischemia, Mac-1 positive microglial cells became activated and increase expression of Mac-1 [23, 50]. By 6 h after MCAO, there was a dramatic increase in vessel-associated PDGFRα phosphorylation in wild-type mice, which was not apparent in Mac-1^−/−^ mice. Interestingly, this rise in PDGFRα phosphorylation correlates very well with the early transient rise in BBB permeability which has been reported to occur between 4 and 8 h after MCAO [35, 37]. Thus, these data suggest that the early transient increase in BBB permeability following MCAO may be mediated by PDGF-CC signaling through the PDGFRα and that the regulation of this signaling is controlled in part by the association of activated microglia with the NVU. This is an intriguing regulatory mechanism as it has been proposed that the NVU may function as a dynamic interface between the innate immune system of the CNS controlled by microglia and the systemic innate and adaptive immune system [38]. We can speculate that the early transient rise in BBB permeability may permit the release to the blood of bioactive signaling molecules such as cytokines or damage-associated molecular pattern molecules (DAMPs) [54] produced by activated microglia and the injured brain tissue.

Our data showing that Mac-1 deficiency reduces BBB permeability and infarct size, together with earlier studies showing that tPA deficiency or its inhibition, or the inhibition of PDGF-CC signaling similarly reduce BBB permeability and infarct size [61, 69, 70] suggest that in the context of MCAO, this early rise in BBB permeability may be a critical factor contributing to infarct expansion and in extreme cases to hemorrhagic conversion. The results presented here also extend our understanding of the potential benefit of interventions targeting LRP1 [51, 69], or Mac-1 [72]. Importantly, our data demonstrating that Mac-1 deficiency dramatically reduces spontaneous ICH associated with late thrombolysis are consistent with our earlier study showing that PDGFRα antagonists can reduce thrombolysis-associated ICH [61, 69, 70] and support the idea that targeting PDGFRα signaling in stroke has the potential to significantly improve the safety of thrombolytic therapy. Toward that goal, the recent phase II clinical trial testing the safety of the PDGFRα antagonist Imatinib in patients treated with intravenous thrombolysis after ischemic stroke has demonstrated that Imatinib is safe following thrombolysis and improves neurological outcomes [66]. If these data are confirmed in a phase III clinical trial, this would be the first new pharmacological treatment for ischemic stroke since the approval of tPA by the FDA in 1996.

Finally, it is also interesting to speculate that if the increased permeability associated with PDGFRα signaling in the NVU does act to promote CNS inflammation, then the benefits of inhibiting this pathway may be wider than just in ischemic stroke. In this regard, it is interesting to note that previous studies have demonstrated a significant benefit of PDGFRα antagonists in several CNS pathologies and injuries, including ischemic and hemorrhagic stroke [61, 71], spinal cord injury [2, 33], experimental autoimmune encephalomyelitis (a model for multiple sclerosis) [3], traumatic brain injury [63], and amyotrophic lateral sclerosis [40], all disorders where neuroinflammation is thought to play a role. In addition, recent studies in animal models of Alzheimer’s [41] and Parkinson’s disease [27] have shown significant benefit from treatment with the related PDGFRα antagonists Nilotinib and Bosutinib. However, in both these studies, it was suggested that the benefit was mediated by inhibition of the c-Abl tyrosine kinase, although the effects of these agents on the BBB or PDGFRα signaling were not examined.

In summary, our data provide both in vitro and in vivo evidence that Mac-1 acting together with LRP1 facilitates PDGF-CC activation by tPA in the NVU. The requirement of both Mac-1 and LRP1 for efficient activation of PDGF-CC by tPA provides a novel mechanism that helps limit PDGFRα signaling in the NVU. This restriction underscores the importance of the tight regulation of PDGF-CC activation in the brain, implying that aberrant activation of this pathway may have significant harmful consequences such as the loss of BBB control. We suggest that PDGF-CC may act to promote communication between the innate immune system of the CNS and the peripheral immune system, thus allowing these two systems to function in a coordinated manner to promote wound healing in the brain following injury. However, prolonged or over-activation of PDGF-CC, e.g., following tPA administration, may also exacerbate neurological diseases.

## Electronic supplementary material

Below is the link to the electronic supplementary material.

Supplementary material 1 (PDF 2465 kb)

## Abbreviations

BBB: Blood–brain barrier
tPA: Tissue-type plasminogen activator
Mac-1: Macrophage-1 antigen
LRP1: Low-density lipoprotein receptor-related protein 1
