# Supplementary material for: Microglial-mediated PDGF-CC activation increases cerebrovascular permeability during ischemic stroke
Source: Acta Neuropathol. 2017 Jul 19;134(4):585–604. doi: 10.1007/s00401-017-1749-z (PMC5587628; doi:10.1007/s00401-017-1749-z)
Supplement: Supplementary file 1 — Supplementary material 1 (PDF 2465 kb) [file 401_2017_1749_MOESM1_ESM.pdf]

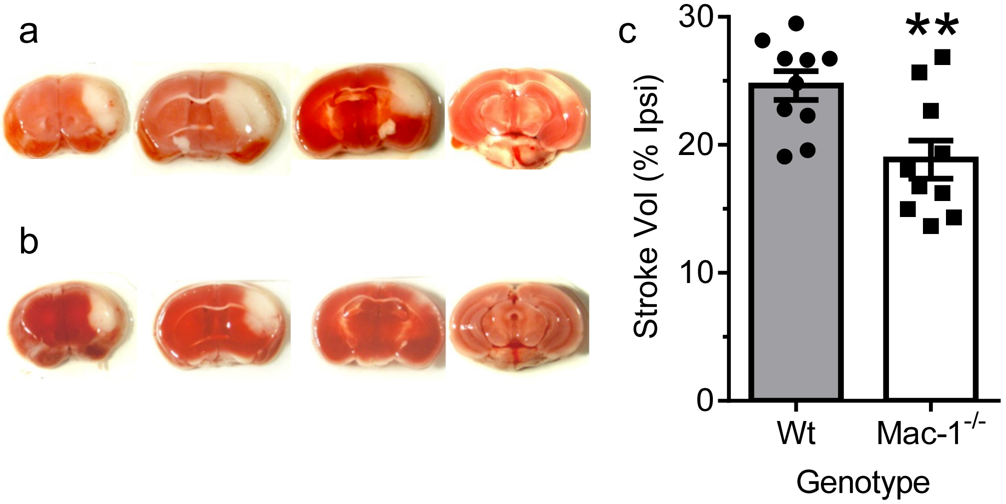

Figure S1. Mac-1 deficiency reduces infarct size after MCAO. a-b) Wild-type and Mac-1$^{-/-}$ mice were subjected to MCAO and seventy two hours later, brains were sectioned and stained with TTC. Representative images were shown in wild-type (a) and Mac-1$^{-/-}$ mice (b). **c**) Quantification of infarct size 72 hours after MCAO in wild-type or Mac-1$^{-/-}$ mice (n = 8-10). Errors represent S.E.M and ** p<0.01 (unpaired two-tail t-test).

Online Resource 1

Title: Microglial mediated PDGF-CC activation increases cerebrovascular permeability during ischemic stroke

Journal: Acta Neuropathologica

Authors: Enming Joseph Su, Chunzhang Cao, Linda Fredriksson, Ingrid Nilsson, Christina Stefanitsch, Tamara K. Stevenson, Juanjuan Zhao, Margret Ragsdale, Yu-Yo Sun, Manuel Yepes, Chia-Yi Kuan, Ulf Eriksson, Dudley K Strickland, Daniel A Lawrence, and Li Zhang
Corresponding Authors:
Dr. Lawrence: University of Michigan Medical School, E-mail: dlawrenc@umich.edu
Dr. Zhang: University of Maryland School of Medicine, lizhang@som.umaryland.edu

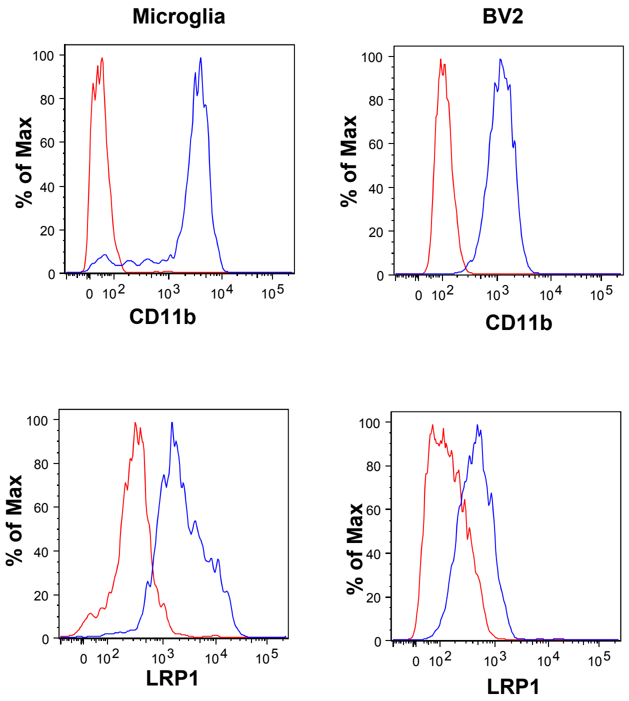

Figure S2 (related to Fig. 1). Primary microglia and BV2 both express LRP1 and CD11b on their cell surface. Cortices from 1 to 3-day old WT mice were dissected and then treated with 0.4% trypsin for 20 min at 37°C. Cells were plated onto poly-D-lysine-coated 75-cm^2 tissue culture flasks and cultured in 10% FBS in DMEM. After 2-3 weeks, microglia were removed, washed and then incubated with rabbit anti-LRP1 (Ab 2629), rat anti-CD11b (M1/70) or control IgG, followed by FITC conjugates of their respective secondary antibody. Expression of LRP1 or CD11b was detected by flow cytometry. Red line: control IgG; Blue line: CD11b or LRP1

Online Resource 2
Title: Microglial mediated PDGF-CC activation increases cerebrovascular permeability during ischemic stroke
Journal: Acta Neuropathologica
Authors: Enming Joseph Su, Chunzhang Cao, Linda Fredriksson, Ingrid Nilsson, Christina Stefanitsch, Tamara K. Stevenson, Juanjuan Zhao, Margret Ragsdale, Yu-Yo Sun, Manuel Yepes, Chia-Yi Kuan, Ulf Eriksson, Dudley K Strickland, Daniel A Lawrence, and Li Zhang
Corresponding Authors:
Dr. Lawrence: University of Michigan Medical School, E-mail: dlawrenc@umich.edu
Dr. Zhang: University of Maryland School of Medicine, lizhang@som.umaryland.edu

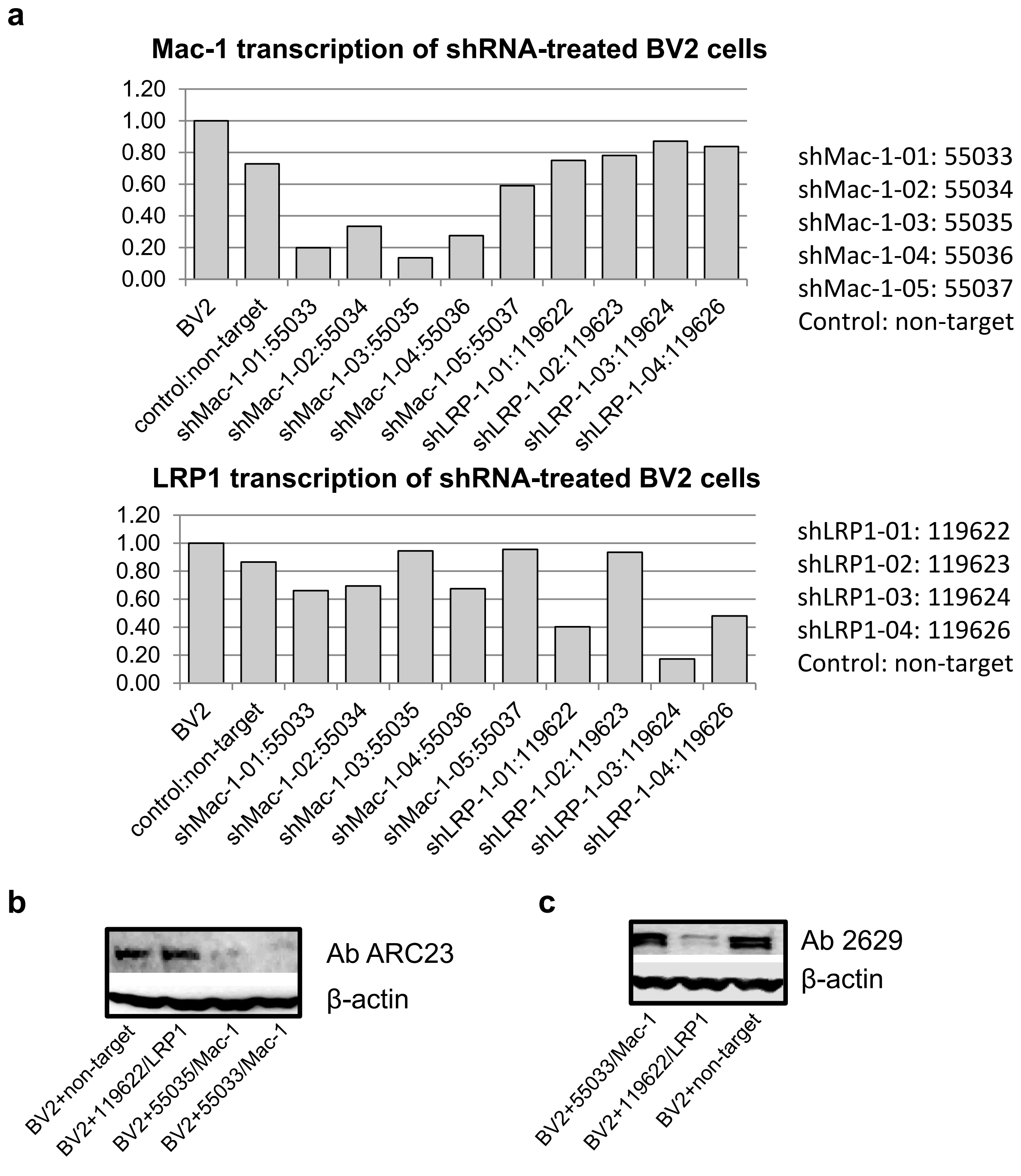

Figure S3 (related to Fig. 2b-2c). Specific shRNAs inhibited expression of Mac-1 or LRP1 without cross reactivity. a) BV2 cells were infected with lentivirus encoding a control shRNA, shRNA for Mac-1 or shRNA for LRP1 and the specific knockdown of Mac-1 and LRP1 expression by BV2 cells was assessed by qRT-PCR. **b**) Immunoblotting with an anti-Mac-1 antibody ARC23 of samples from shRNA treated BV2 cells. **c**) Immunoblotting with an anti-LRP1 antibody 2629 of samples from shRNA treated BV2 cells.

Online Resource 3
Title: Microglial mediated PDGF-CC activation increases cerebrovascular permeability during ischemic stroke
Journal: Acta Neuropathologica
Authors: Enming Joseph Su, Chunzhang Cao, Linda Fredriksson, Ingrid Nilsson, Christina Stefanitsch, Tamara K. Stevenson, Juanjuan Zhao, Margret Ragsdale, Yu-Yo Sun, Manuel Yepes, Chia-Yi Kuan, Ulf Eriksson, Dudley K Strickland, Daniel A Lawrence, and Li Zhang
Corresponding Authors:
Dr. Lawrence: University of Michigan Medical School, E-mail: dlawrenc@umich.edu
Dr. Zhang: University of Maryland School of Medicine, lizhang@som.umaryland.edu

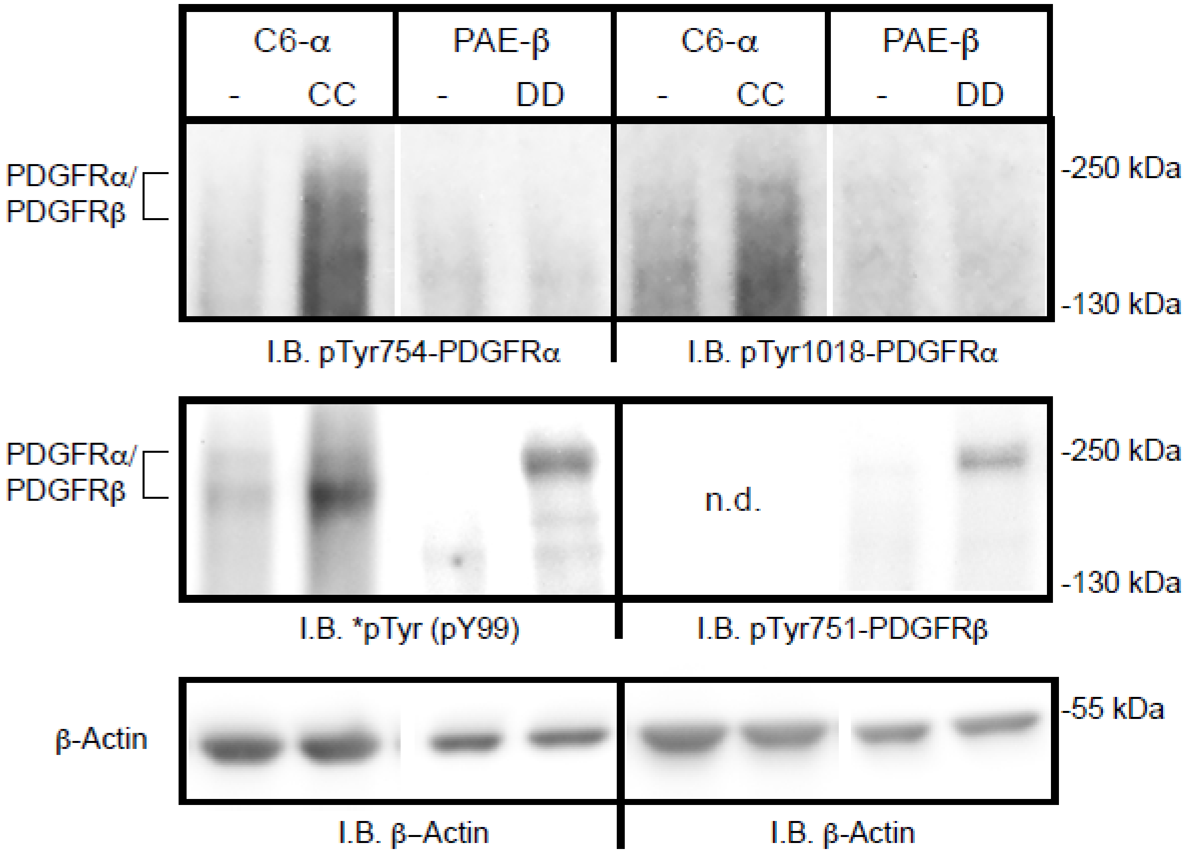

Figure S4 (related to Fig. 7). Target specificity of phospho-specific PDGFRα antibodies. PDGFRα expressing C6 cells (C6-α) and PDGFRβ expressing PAE cells (PAE-β) were acutely stimulated with active PDGF-CC (CC) or PDGF-DD (DD) to selectively induce PDGFRα and PDGFRβ phosphorylation, respectively. Western blot with the pTyr754 and pTyr1018 specific PDGFRα antibodies show selectivity towards phosphorylated PDGFRα in C6-α lysates, but not towards PDGFRβ in PAE-β lysates (upper panels). PDGFRs migrate around 190 kDa and presents with several bands, representing different glycosylation levels. General pTyr (*pTyr pY99) and pTyr751 PDGFRβ antibodies confirmed phosphorylation status of PDGFRα and –β in stimulated cells (middle panels). Lower panels demonstrate equal loading using a β-Actin antibody.

Online Resource 4
Title: Microglial mediated PDGF-CC activation increases cerebrovascular permeability during ischemic stroke
Journal: Acta Neuropathologica

Authors: Enming Joseph Su, Chunzhang Cao, Linda Fredriksson, Ingrid Nilsson, Christina Stefanitsch, Tamara K. Stevenson, Juanjuan Zhao, Margret Ragsdale, Yu-Yo Sun, Manuel Yepes, Chia-Yi Kuan, Ulf Eriksson, Dudley K Strickland, Daniel A Lawrence, and Li Zhang
Corresponding Authors:
Dr. Lawrence: University of Michigan Medical School, E-mail: dlawrenc@umich.edu
Dr. Zhang: University of Maryland School of Medicine, lizhang@som.umaryland.edu

a

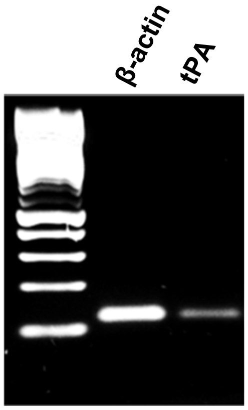

b

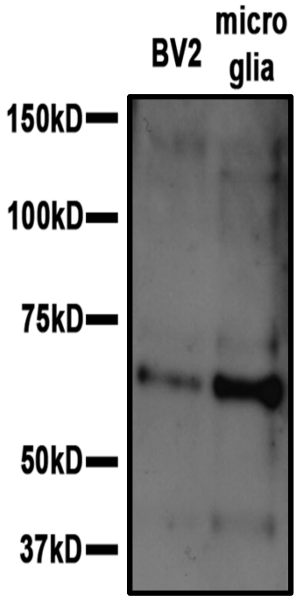

c

DNA primers for RT-PCR:

Primer Name	Protein	Gene Symbol	Sequence (5′ to 3′)	Length	Species	PCR size
NM_008872_L1	Plat	NM_008872	GAGCCAACGCAGACAACTTA	20	Mouse	115bp
NM_008872_R1	Plat	NM_008872	GCACTGAGTGGCATTGTACC	20	Mouse	115bp
NM_007393_LX1	Actb	NM_007393	GGTCATCACTATTGGCAACG	20	Mouse	133bp
NM_007393_RX1	Actb	NM_007393	ACGGATGTCAACGTCACACT	20	Mouse	133bp

Figure S5 (related to Fig. 1). Microglial BV2 cells express tPA. a) RT-PCR. Total RNA was extracted from BV2 cells using Absolutely RNA miniprep kit (Sigma-Aldrich) according to the manufactures' instructions. One microgram of total RNA was used for cDNA synthesis using Superscript II and random hexamers (Thermo Fisher). RT-PCR was performed using their corresponding primers (Panel C below) with the following settings: activation of the AmpliTaq Gold Polymerase at 94 °C for 3 min, 30 cycles of 94 °C for 30 s, 62 °C for 30 s and 72 °C for 30 s. The RT-PCR products were separated on 2% agarose gel. A Roche DNA molecular marker XIV was used as DNA size markers. **b**) Immunoblotting. BV2 cells and murine primary microglia (prepared from wildtype C57BL6 neonatal mice) were lyzed in 1XRIPA buffer (Thermo Fisher). The cell lysates were separated on 10% SDS-PAGE, transferred to PVDF membranes, and subjected to western blot using a rabbit anti-tPA antibody (Sigma-Aldrich). **c**) Primers for RT-PCR.

Authors: Enming Joseph Su, Chunzhang Cao, Linda Fredriksson, Ingrid Nilsson, Christina Stefanitsch, Tamara K. Stevenson, Juanjuan Zhao, Margret Ragsdale, Yu-Yo Sun, Manuel Yepes, Chia-Yi Kuan, Ulf Eriksson, Dudley K Strickland, Daniel A Lawrence, and Li Zhang
Corresponding Authors:
Dr. Lawrence: University of Michigan Medical School, E-mail: dlawrenc@umich.edu
Dr. Zhang: University of Maryland School of Medicine, lizhang@som.umaryland.edu

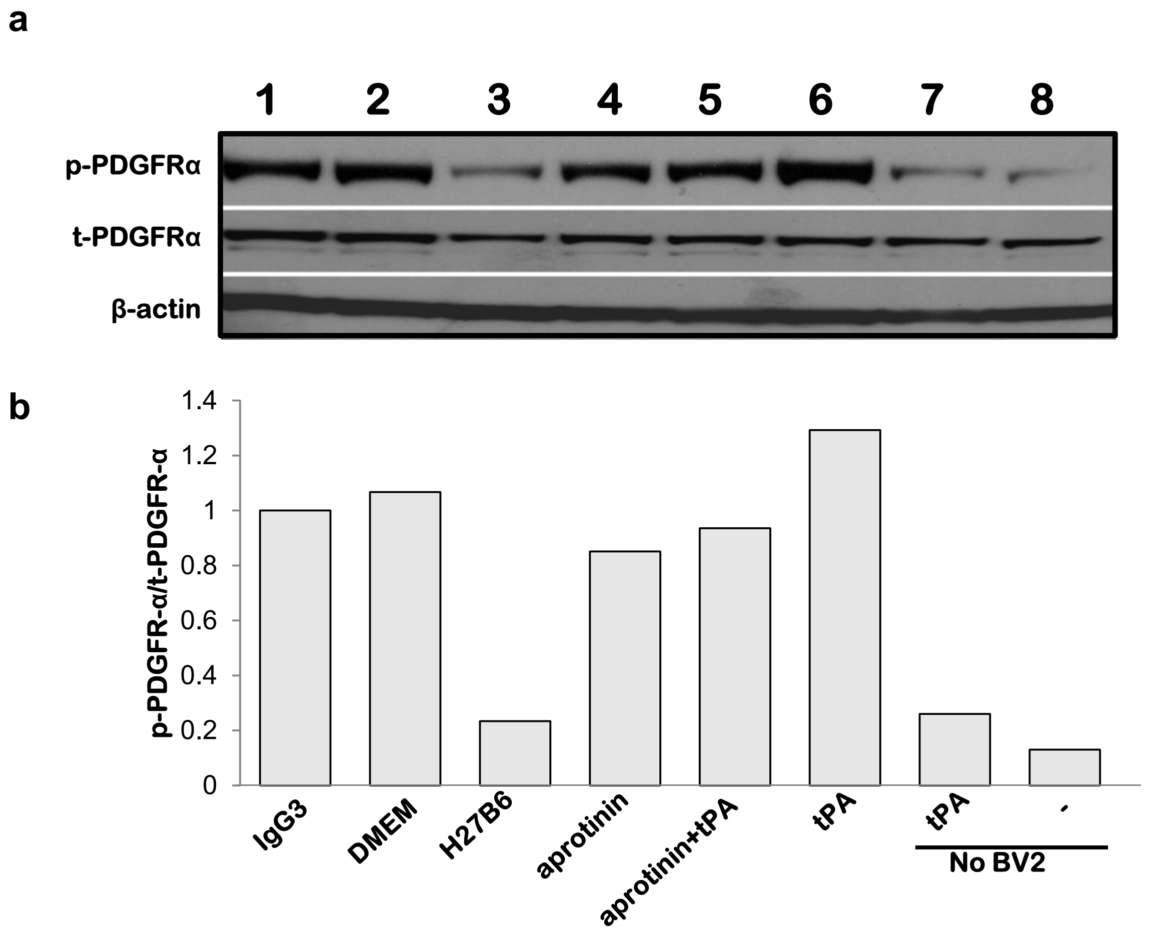

Figure S6 (related to Fig. 2a). Activation of PDGF-CC by microglia is dependent on tPA but not plasmin. a) Latent PDGF-CC was incubated with BV2 cells in the presence of a control IgG3 (lane 1), DMEM (lane 2), an anti-murine tPA mAb H27B6 (lane 3), a plasmin inhibitor aprotinin (lane 4), tPA plus aprotinin (lane 5) or additional tPA (lane 6). As controls, latent PDGF-CC was incubated in the absence of BV2 cells with (lane 7) or without (lane 8) tPA. The generation of active PDGF-CC was detected by its ability to stimulate PDGFRα phosphorylation (p-PDGFRα). Total PDGFRα (t-PDGFRα) was used as a loading control. **b**) Quantification of the data in panel a.

Online Resource 6
Title: Microglial mediated PDGF-CC activation increases cerebrovascular permeability during ischemic stroke
Journal: Acta Neuropathologica
Authors: Enming Joseph Su, Chunzhang Cao, Linda Fredriksson, Ingrid Nilsson, Christina Stefanitsch, Tamara K. Stevenson, Juanjuan Zhao, Margret Ragsdale, Yu-Yo Sun, Manuel Yepes, Chia-Yi Kuan, Ulf Eriksson, Dudley K Strickland, Daniel A Lawrence, and Li Zhang
Corresponding Authors:
Dr. Lawrence: University of Michigan Medical School, E-mail: dlawrenc@umich.edu
Dr. Zhang: University of Maryland School of Medicine, lizhang@som.umaryland.edu

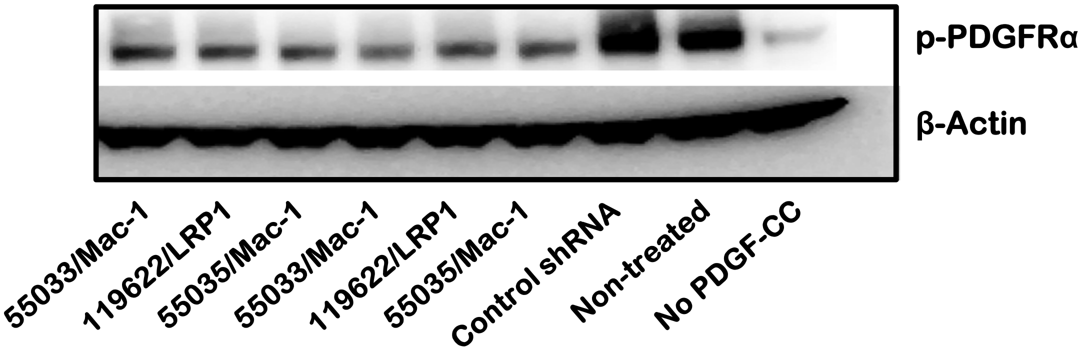

Figure S7 (related to Fig. 2b). shRNA knockdown of Mac-1 or LRP1 expression inhibited the ability of BV2 cells to support PDGF-CC activation. Latent PDGF-CC was incubated with BV2 or shRNA-treated BV2. Generation of active PDGF-CC was determined by its ability to stimulate PDGFRα phosphorylation on PAE cells.

Online Resource 7
Title: Microglial mediated PDGF-CC activation increases cerebrovascular permeability during ischemic stroke
Journal: Acta Neuropathologica
Authors: Enming Joseph Su, Chunzhang Cao, Linda Fredriksson, Ingrid Nilsson, Christina Stefanitsch, Tamara K. Stevenson, Juanjuan Zhao, Margret Ragsdale, Yu-Yo Sun, Manuel Yepes, Chia-Yi Kuan, Ulf Eriksson, Dudley K Strickland, Daniel A Lawrence, and Li Zhang
Corresponding Authors:
Dr. Lawrence: University of Michigan Medical School, E-mail: dlawrenc@umich.edu
Dr. Zhang: University of Maryland School of Medicine, lizhang@som.umaryland.edu

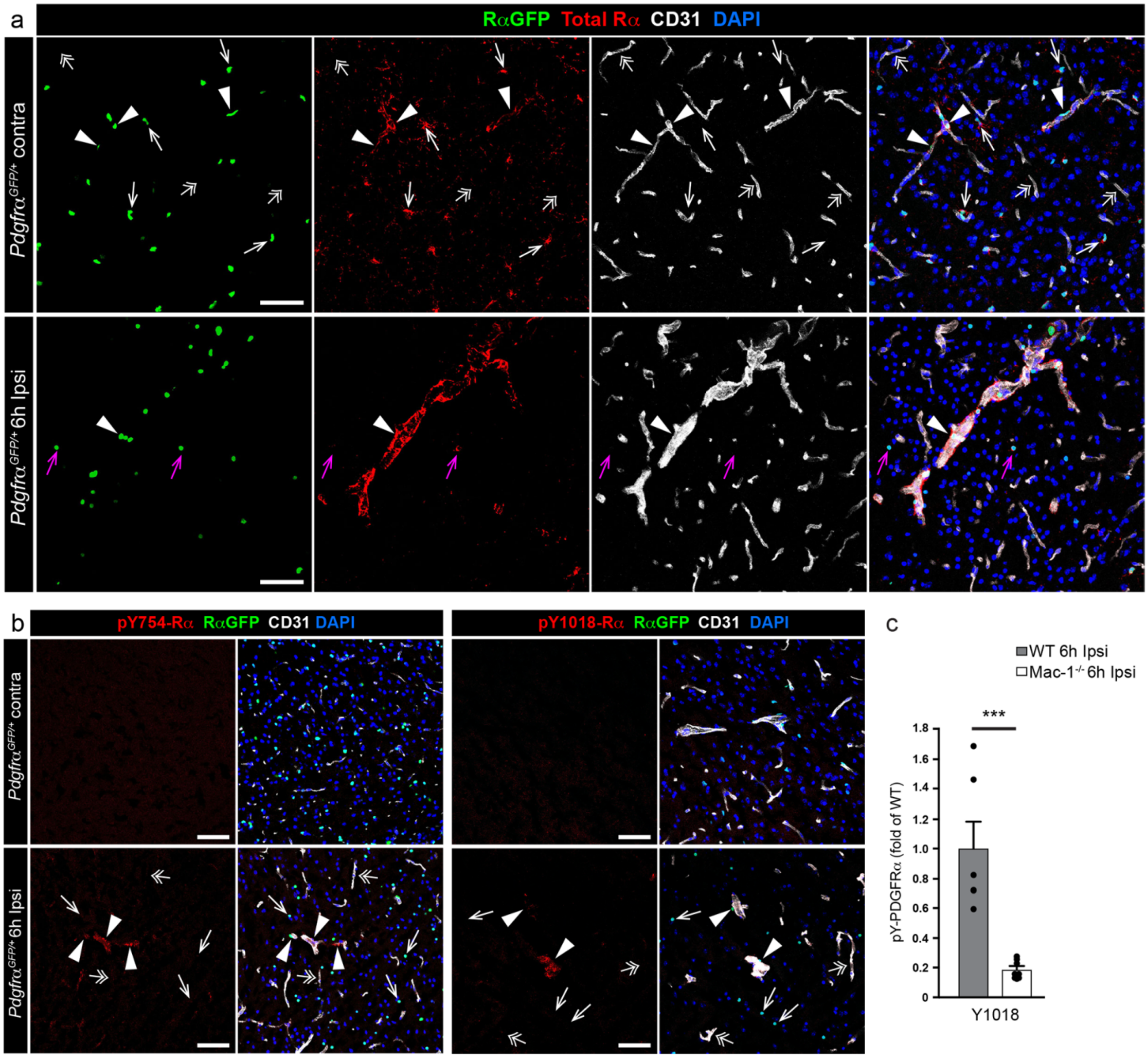

Figure S8 (related to Fig. 7). PDGFRα activation in the NVU after MCAO. a) PDGFRα immunostaining co-localizes to GFP positive nuclei both in nonvascular (arrows) and arteriolar-associated cells (arrowheads) in the contralateral hemisphere of PdgfrαGFP/+ mice 6 hours after MCAO. No GFP reporter or PDGFRα immunoreactivity was found around capillary-sized vessels (double arrows). In the ischemic border (ipsi), PDGFRα immunoreactivity was decreased in nonvascular cells (still expressing GFP, magenta arrows), whereas the vessel-associated PDGFRα increased correspondingly (arrowheads). **b)** In situ activation of PDGFRα was determined by immunostaining with phosphotyrosine-specific PDGFRα antibodies (pY754 and pY1018, respectively). No activation of PDGFRα was observed in contralateral hemisphere of PdgfrαGFP/+ mice 6 hours after MCAO using either antibody. In the ischemic border, robust PDGFRα activation was evident at 6 hours post-MCAO, specifically around vessels invested with GFP positive nuclei (arrowheads), but not in nonvascular GFP positive cells (arrows). **c)** Quantification of pY1018-PDGFRα immunoreactivity in WT (n=6) and Mac-1$^{-/-}$ (n=8) mice. Data display individual data points and group means ± S.E.M. Statistical evaluation was performed using unpaired t-test and *** p<0.001. Cell nuclei were visualized by DAPI (blue). The pictures are representative images from the respective staining and were captured in the ischemic penumbra or the contralateral cortex. The images display the maximum intensity projections generated from confocal Z-stacks. Scale bars, 50 μm.

Online Resource 8

Title: Microglial mediated PDGF-CC activation increases cerebrovascular permeability during ischemic stroke

Journal: Acta Neuropathologica

Authors: Enming Joseph Su, Chunzhang Cao, Linda Fredriksson, Ingrid Nilsson, Christina Stefanitsch, Tamara K. Stevenson, Juanjuan Zhao, Margret Ragsdale, Yu-Yo Sun, Manuel Yepes, Chia-Yi Kuan, Ulf Eriksson, Dudley K Strickland, Daniel A Lawrence, and Li Zhang

Corresponding Authors:

Dr. Lawrence: University of Michigan Medical School, E-mail: dlawrenc@umich.edu

Dr. Zhang: University of Maryland School of Medicine, lizhang@som.umaryland.edu

Supplementary Table 1 (related to Fig. 4)	Statistical Comparisons		
**two-way ANOVA with Fisher's LSD**	Mean Diff.	95% CI of diff.	P Value
**WT:PBS vs. WT:tPA**	-0.407	-0.55 to -0.263	<0.001
**WT:PBS vs. WT:PDGF-CC**	-0.358	-0.502 to -0.215	<0.001
**WT:PBS vs. Mac-1-/-:PBS**	0.02	-0.123 to 0.163	0.778
**WT:PBS vs. Mac-1-/-:tPA**	-0.0633	-0.207 to 0.0801	0.374
**WT:PBS vs. Mac-1-/-:PDGF-CC**	-0.277	-0.42 to -0.133	<0.001
**WT:tPA vs. WT:PDGF-CC**	0.0483	-0.0951 to 0.192	0.496
**WT:tPA vs. Mac-1-/-:PBS**	0.427	0.283 to 0.57	<0.001
**WT:tPA vs. Mac-1-/-:tPA**	0.343	0.2 to 0.487	<0.001
**WT:tPA vs. Mac-1-/-:PDGF-CC**	0.13	-0.0134 to 0.273	0.074
**WT:PDGF-CC vs. Mac-1-/-:PBS**	0.378	0.235 to 0.522	<0.001
**WT:PDGF-CC vs. Mac-1-/-:tPA**	0.295	0.152 to 0.438	<0.001
**WT:PDGF-CC vs. Mac-1-/-:PDGF-CC**	0.0817	-0.0617 to 0.225	0.254
**Mac-1-/-:PBS vs. Mac-1-/-:tPA**	-0.0833	-0.227 to 0.0601	0.245
**Mac-1-/-:PBS vs. Mac-1-/-:PDGF-CC**	-0.297	-0.44 to -0.153	<0.001
**Mac-1-/-:tPA vs. Mac-1-/-:PDGF-CC**	-0.213	-0.357 to -0.0699	0.005

Online Resource 9

Title: Microglial mediated PDGF-CC activation increases cerebrovascular permeability during ischemic stroke
Journal: Acta Neuropathologica
Authors: Enming Joseph Su, Chunzhang Cao, Linda Fredriksson, Ingrid Nilsson, Christina Stefanitsch, Tamara K. Stevenson, Juanjuan Zhao, Margret Ragsdale, Yu-Yo Sun, Manuel Yepes, Chia-Yi Kuan, Ulf Eriksson, Dudley K Strickland, Daniel A Lawrence, and Li Zhang
Corresponding Authors:
Dr. Lawrence: University of Michigan Medical School, E-mail: dlawrenc@umich.edu
Dr. Zhang: University of Maryland School of Medicine, lizhang@som.umaryland.edu

**Supplementary Table 2 (related to Fig. 8b)**	**Statistical Comparisons**		
**two-way ANOVA with Fisher's LSD**	Mean Diff.	95% CI of diff.	P Value
**0 hrs:Microglia vs. 0 hrs:Monocyte**	73.5	18.89 to 128.1	0.0125
**0 hrs:Microglia vs. 6 hrs:Microglia**	-48.5	-98.35 to 1.352	0.0556
**0 hrs:Microglia vs. 6 hrs:Monocyte**	68.17	18.31 to 118	0.0115
**0 hrs:Microglia vs. 24:Microglia**	-91.25	-138.5 to -43.96	0.0012
**0 hrs:Microglia vs. 24:Monocyte**	15.75	-31.54 to 63.04	0.4820
**0 hrs:Monocyte vs. 6 hrs:Microglia**	-122	-171.9 to -72.15	0.0002
**0 hrs:Monocyte vs. 6 hrs:Monocyte**	-5.333	-55.19 to 44.52	0.8196
**0 hrs:Monocyte vs. 24:Microglia**	-164.8	-212 to -117.5	<0.0001
**0 hrs:Monocyte vs. 24:Monocyte**	-57.75	-105 to -10.46	0.0208
**6 hrs:Microglia vs. 6 hrs:Monocyte**	116.7	72.08 to 161.3	<0.0001
**6 hrs:Microglia vs. 24:Microglia**	-42.75	-84.46 to -1.041	0.0454
**6 hrs:Microglia vs. 24:Monocyte**	64.25	22.54 to 106	0.0057
**6 hrs:Monocyte vs. 24:Microglia**	-159.4	-201.1 to -117.7	<0.0001
**6 hrs:Monocyte vs. 24:Monocyte**	-52.42	-94.13 to -10.71	0.0180
**24:Microglia vs. 24:Monocyte**	107	68.38 to 145.6	<0.0001

Online Resource 10

Title: Microglial mediated PDGF-CC activation increases cerebrovascular permeability during ischemic stroke
Journal: Acta Neuropathologica
Authors: Enming Joseph Su, Chunzhang Cao, Linda Fredriksson, Ingrid Nilsson, Christina Stefanitsch, Tamara K. Stevenson, Juanjuan Zhao, Margret Ragsdale, Yu-Yo Sun, Manuel Yepes, Chia-Yi Kuan, Ulf Eriksson, Dudley K Strickland, Daniel A Lawrence, and Li Zhang
Corresponding Authors:
Dr. Lawrence: University of Michigan Medical School, E-mail: dlawrenc@umich.edu
Dr. Zhang: University of Maryland School of Medicine, lizhang@som.umaryland.edu
